# Isolation of two rare *N-*glycosides from *Ginkgo biloba* and their anti-inflammatory activities

**DOI:** 10.1038/s41598-020-62884-1

**Published:** 2020-04-07

**Authors:** Jin-Tang Cheng, Cong Guo, Wen-Jin Cui, Qing Zhang, Shu-Hui Wang, Qing-He Zhao, De-Wen Liu, Jun Zhang, Sha Chen, Chang Chen, Yan Liu, Zheng-Hong Pan, An Liu

**Affiliations:** 1 Institute of Chinese Materia Medica, China Academy of Chinese Medical Sciences, Beijing, 100700 China; 2 Guangxi Key Laboratory of Functional Phytochemicals Research and Utilization, Guangxi Institute of Botany, Guangxi Zhuang Autonomous Region and Chinese Academy of Sciences, Guilin, 541006 China

**Keywords:** Drug discovery and development, Secondary metabolism

## Abstract

Two rare *N*-β-D-glucopyranosyl-1*H*-indole-3-acetic acid conjugates, *N*-[2-(1-β-D-glucopyranosyl)-1*H*-indol-3-yl)acetyl]-L-glutamic acid (**1**) and *N*-[2-(1-β-D-glucopyranosyl)-1*H*-indol-3-yl)acetyl]-L-aspartic acid (**2**) were isolated from *Ginkgo biloba*. The structures were elucidated by analyses of HRMS and NMR spectroscopic data. In addition, a simplified and efficient synthetic route for compounds **1** and **2** is also disclosed to determine the absolute configurations of them. This concise syntheses of compounds **1** and **2** may facilitate studies of the biology of this type alkaloids. Compounds **1** and **2** were also tested for their cytotoxic and anti-inflammatory activities. The biological evaluation showed that compounds **1** and **2** led to the decrease of interleukin (IL)-6, nitric oxide synthase (iNOS) and cyclooxygenase (COX)-2 at mRNA level in lipopolysaccharide (LPS)-stimulated murine macrophage RAW264.7 cells.

## Introduction

*Ginkgo biloba* L., one of the most well-known medicinal plants worldwide, is considered as a living fossil due to its survival over millions of years^1^. Pharmacological studies have shown that extracts from its leaves and seeds exhibit antiparasitic, antifungal, antibacterial and antiviral activities. *G. biloba* extracts have also found broad applications in the treatment of cognitive diseases^2–5^. Since the discovery of ginkgolides A-C, a lot of phytochemical and pharmaceutical studies have been conducted on the *G. biloba* leaves^6–8^. It is well known that *G. biloba* leaf extracts, mainly composed of ginkgolides, flavonoids and phenolic compounds, are frequently used in the treatment of cardiovascular, cerebrovascular, and neurological diseases^9,10^.

**Figure 1:**
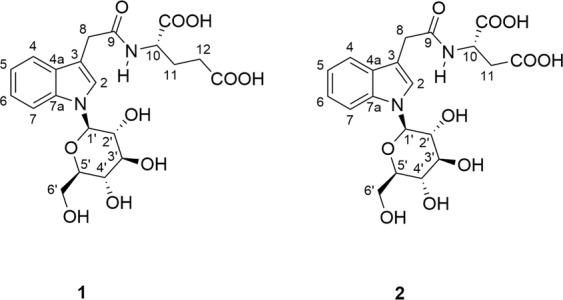
The structures of compounds **1** and **2**.

The dried seeds of *G. biloba* (called “baiguo” in Chinese), have been reported in the Chinese Pharmacopoeia as effective treatments for cough, asthma, enuresis, alcohol misuse, pyogenic skin infections and worm infestations in the intestinal tract^11,12^. In contrast to extensive studies on *G. biloba* leaves, the seeds of *G. biloba* have received much less attention. In order to find structurally interesting and bioactive components from Ginkgo seeds and provide a better understanding of their functions, we conducted a phytochemical investigation of Ginkgo seeds. As a result, two *N*-β-D-glucopyranosyl-1*H*-indole-3-acetic acid conjugates were isolated from the title plant and the concise syntheses of **1** and **2** were also enclosed to determine the absolute configurations of compounds **1** and **2** (Fig. 1). Notably, compounds **1** and **2** are naturally isolated *N*-glycosides, which are rarely found in natural products. To the best of our knowledge, only very few examples have been reported previouly^13–17^. Although compounds **1** and **2** were detected in an alkaline hydrolysate of rice extract using liquid chromatography-electrospray ionization-tandem mass spectrometry (LC-ESI-MS/MS)^18^, there are no examples of their isolation, absolute configurations determination and bioactivities. Reported herein are the structural identification, concise syntheses and bioactivities of them.

## Results and discussion

**Figure 2:**
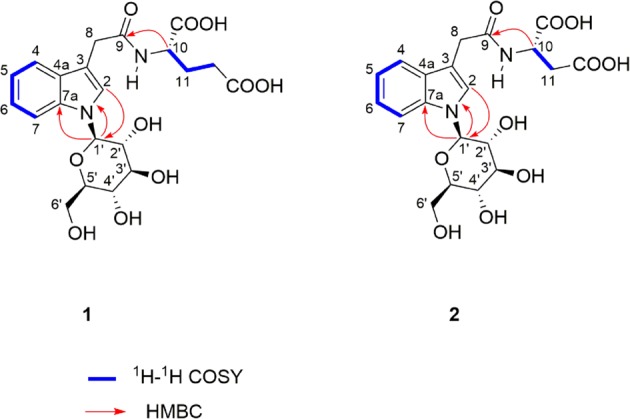
Key COSY and HMBC correlations of compounds **1** and **2**.

Ginkgoside A (**1**), a yellow-brownish solid, was determined to have the molecular formula C_21_H_26_N_2_O_10_ from its HRESIMS data at *m/z* 467.1662 (calc. 467.1660). The ^1^H NMR data (Table 1) displayed an *ortho*-substituted aromatic ring (*δ*_H_ 7.53, 2H, dd, *J* = 8.1, 3.2 Hz; 7.17, 1 H, t, *J* = 7.1 Hz; 7.07, 1 H, t, *J* = 6.8 Hz), an isolated aromatic proton (*δ*_H_ 7.37, 1 H, s), a glucopyranosyl moiety (*δ*_H_ 5.34, 3.90, 3.58, 3.50, 3.55, 3.86, 3.69), a deshielded methine (*δ*_H_ 4.43, 1 H, m), as well as three methylenes (*δ*_H_ 3.71, 2 H, s; 2.33, 2 H, brs; 2.17, 1 H, d, *J* = 6.8 Hz; 1.93, 1 H, m). The ^13^C NMR and HSQC spectra exhibited the presence of twenty one carbon signals attributable to six quaternary carbons (*δ*_C_ 176.9, 175.7, 174.7, 138.7, 129.7, 110.7), eleven methines, and four methylenes (*δ*_C_ 63.1, 33.5, 31.5, 28.2). An 3-indole acetic acid moiety in compound **1** could be easily deduced from the characteristic proton signals (*δ*_H_ 7.53, 2 H, dd, *J* = 8.1, 3.2 Hz; 7.37, 1 H, s; 7.17, 1 H, t, *J* = 7.1 Hz; 7.07, 1 H, t, *J* = 6.8 Hz; 3.71, 2 H, s), together with the corresponding signals displayed in its ^13^C NMR spectrum^13^. The 2D NMR analysis (Fig. 2) further supported the above conclusion. In addition, comparing the characteristic ^13^C NMR data in the region of *δ*_C_ 86.9–63.1 (Table 1) with the reported structure in the literature revealed the presence of an *N*-glucose moiety, showing key correlations of H-1′/C-2, H-1′/C-7a, H-2/C-1′ in its HMBC spectrum^14^.

**Table 1:** ^1^H NMR (600 MHz) and ^13^C NMR (150 MHz) spectra of compounds **1** and **2** in methanol-*d*_4_ (*δ* in ppm, *J* in Hz).

NO.	Ginkgoside A (1)		Ginkgoside B (2)	
	*δ*_C_, type	*δ*_H_, mult (*J* in Hz)	*δ*_C_, type	*δ*_H_, mult (*J* in Hz)
2	125.8, CH	7.37, s	126.4, CH	7.36, s
3	110.7, C		110.4, C	
4a	129.7, C		129.8, C	
4	120.0, CH	7.53, dd (8.1, 3.2)	120.0, CH	7.62, dd (8.0, 3.2)
5	121.2, CH	7.07, t (6.8)	121.2, CH	7.07, t (7.4)
6	123.2, CH	7.17, t (7.1)	123.6, CH	7.17, t (7.7)
7	111.7, CH	7.53, dd (8.1, 3.2)	111.6, CH	7.62, dd (8.0, 3.2)
7a	138.7, C		138.7, C	
8	33.5, CH_2_	3.71, s	33.5, CH_2_	3.70, s
9	174.7, C		174.3, C	
10	53.6, CH	4.43, m	49.1, CH	4.75, m
11	28.2, CH_2_	2.17, d (6.8) 1.93, m	37.0, CH_2_	2.82, m
12	31.5, CH_2_	2.33, brs		
COOH (1)	175.7, C		174.4, C	
COOH (2)	176.9, C		174.5, C	
1′	86.9, CH	5.43, d (8.8)	86.7, CH	5.43, d (9.0)
2′	73.9, CH	3.90, m	73.9, CH	3.92, dt (8.9, 1.7)
3′	79.0, CH	3.58, m	79.3, CH	3.61, m
4′	71.7, CH	3.50, t (9.0)	71.3, CH	3.51, m
5′	80.7, CH	3.55, m	80.4, CH	3.56, m
6′	63.1, CH_2_	3.86, m 3.69, overlap	62.8, CH_2_	3.86, dd (12.1, 1.6) 3.70, overlap

The large coupling constant (*J* = 8.8 Hz) of the anomeric carbon indicated a β-orientation of H-1′. Analysis of the remaining NMR and HRESIMS data established the presence of a glutamic acid fragment condensed with the indole acetic acid moiety through an amide bond. Thus, the planar structure of **1** was characterized as shown in Fig. 2.

**Figure 3:**
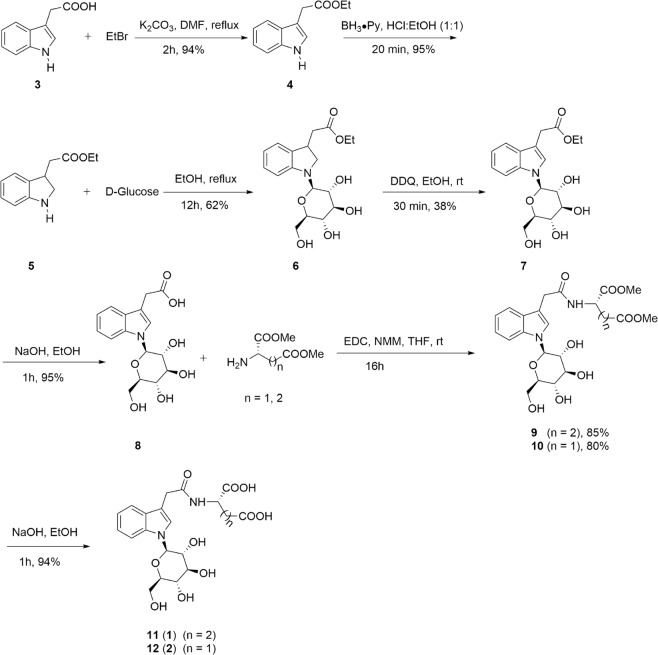
Synthesis of compounds **1** and **2**.

Ginkgoside B (**2**) was isolated as a yellow-brownish solid, and its molecular formula was deduced to be C_20_H_24_N_2_O_10_ based on its positive HRESIMS data at *m/z* 453.1510 (calc. 453.1504). The ^1^H NMR and ^13^C NMR spectra of compound **2** had an overall similarity with those of compound **1**, except for the absence of a CH_2_ group. Careful examination of its NMR data led to the conclusion that the glutamic acid residue was replaced by an aspartic acid residue in compound **2**. However, there were no solid correlations from their ROESY spectra to assign the absolute configurations of the amino acid fragments in compounds **1** and **2**. Therefore, in order to ascertain their absolute configurations, compounds **1** and **2** were synthesized as shown in Fig. 3 via modification of a literature procedure^18,19^.

The synthesis commenced with preparation of IAA ethyl ester (**4**) using indole acetic acid (IAA, **3**) as the starting material. Reduction of IAA ethyl ester (**4**) employing Py·BH_3_ (Pyridine Borane) solution as the reductant provided 2,3-dihydroIAA-OEt (**5**) in 95% yield. 2,3-dihydroIAA-OEt (**5**) was treated with 1.1 equivalent of D-glucose to yield the 2,3-dihydroIAA-OEt-*N*-Glc (**6**) in 62% yield. 2,3-dichloro-5,6-dicyano-1,4-benzoquinone (DDQ) was employed to oxidize **6** to give IAA-OEt-*N*-Glc (**7**), which was further hydrolyzed to give IAA-*N*-Glc (**8**). IAA-*N*-Glc (**8**) was subsequently condensed with L-glutamic acid methyl ester hydrochloride or L-aspartic acid methyl ester hydrochloride to give *N*-glucoside of IAA-amide (**9** and **10**). Finally, treatment of *N*-glucoside of IAA-amide (**9** and **10**) with 2 M NaOH afforded compounds **11** and **12**, which were the same as compounds **1** and **2** by comparison with their TLC behavior and NMR spectra. The previously reported synthetic route consisted of nine steps, including two acetylation reactions^18^. In our syntheis, the acetylation and deacetylation steps required in previously reported cases could be removed, greatly enhancing its step economy. In addition, replacing the previous reducing agent with Py·BH_3_ (Pyridine Borane) solution made the reaction very clean and didn’t require subsequent purification. Furthermore, when we employed DCC as the activating reagent in the procedure of condensing IAA-*N*-Glc (**8**) with amino acid methyl ester hydrochloride, we found that some of the products would undergo racemization, resulting in a sharp drop in yield (yield: Asp, 38%; Glu, 54% in reported literature). Instead, high yields (yield: Asp, 80%; Glu, 85%) were achieved by using EDC as the reagent in our reaction. The simplified synthetic route furnished compounds **1** and **2** in seven steps with total yields of 16% and 15%, respectively. The concise syntheses of compounds **1** and **2** herein may facilitate studies of the biology of this type alkaloids.

**Figure 4:**
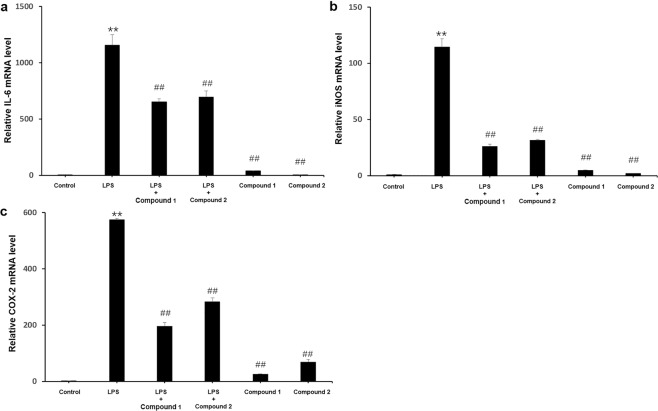
Anti-inflammatory activities of compounds **1** and **2**. (**A**) IL-6, (**B**) iNOS and (**C**) COX-2. **P < 0.01 vs. the control group; ^##^P < 0.01 vs. the LPS-treated group. One-way ANOVA analysis was used to calculate P-values. The bars represent mean ± SD.

Compounds **1** and **2** were evaluated for their cytotoxic activities. Unfortunately, neither of them showed cytotoxic activities against four human cancer cell lines (MCF-7, A-549, HT-29 and HepG2). The anti-inflammatory activities of compounds **1** and **2** were also investigated using lipopolysaccharide(LPS) to stimulate inflammation in RAW264.7. As shown in Fig. 4, compounds **1** and **2** decreased the lipopolysaccharide(LPS)-induced IL-6, iNOS and COX-2 expression at mRNA level.

In conclusion, two *N*-β-D-glucopyranosyl-1*H*-indole-3-acetic acid conjugates were isolated and characterized from the seeds of *G. biloba*. An improved synthetic route is also established here, which is likely to provide an effective means for accessing indole-3-acetic acid conjugates and other analogues. In addition, the results manifested that compounds **1** and **2** might be potent anti-inflammatory agents.

## Methods

### General experimental procedures

Optical rotations were measured with Perkin Elmer/Model-343 digital polarimeter. IR spectra were recorded on a JASCO FT/IR-480 spectrophotometer and reported as wave number (cm^−1^). ^1^H, ^13^C NMR spectra and 2D NMR spectra were recorded on a Bruker Avance III 600 spectrometer. Chemical shifts were reported using TMS as the internal standard. HRESIMS data were obtained on a Bruker Apex IV FTMS spectrometer. Column chromatography (CC) was performed on silica gel (90 − 200 *µ*m; Qingdao Marine Chemical Co. Ltd., Qingdao, People’s Republic of China), and MPLC was performed on a Lisui EZ Purify III System packed with RP-18 silica gel (40–63 *μ*m, Merck, 71 Darmstadt, Germany) columns. Precoated silica gel GF254 plates (Qingdao Marine Chemical Co. Ltd., Qingdao, People’s Republic of China) were used for thin-layer chromatography (TLC). Preparative HPLC was performed on Shimadzu LC-8A equipped with a Shimadzu PRC-ODS(K) column and Agilent 1100 apparatus equipped with a Zorbax SB-C-1875 (Agilent, 9.4 mm × 25 cm) column, respectively. The reagents were purchased from Fisher Scientific at the highest quality and were used without further purification.

### Plant material

The seeds of *G. biloba* were bought from Anguo, Hebei Province, P. R. China in December 2015, and were identified by Dr. Wei Sun (Institute of Chinese Materia Medica, China Academy of Chinese Medical Sciences). A voucher specimen 201512 M) was deposited in the herbarium at the department of medicinal plants, Institute of Chinese Materia Medica, China Academy of Chinese Medical Sciences (Beijing 100700, China).

### Extraction and isolation

The seeds of *G. biloba* (2 kg) were first crushed and then extracted with 40% ethanol under reflux three times (3 h, 2 h, and 1 h, respectively). The resultant extract was resolved in H_2_O and extracted with PE (Petroleum Ether) three times. The water-soluble portion was first subjected to CC (CH_2_Cl_2_:MeOH 10:0 to 0:10) to afford fractions I-V. Fraction IV was chromatographed over repeated silica gel columns (CHCl_3_/MeOH) and finally purified by a C18 HPLC (CH_3_CN/H_2_O/TFA, 18:82:0.1) to afford compounds **1** (54 mg) and **2** (40 mg).

**Ginkgoside A (1)**. Yellow-brownish solid; [α]_D_^25^ + 8.7 (c 0.1, MeOH); IR (KBr) 3421, 2945, 1725, 1384, 1272, 1077 cm^–1^; ^1^H and ^13^C NMR data: see Table 1. HRESIMS *m/z* 467.1662 [M + H]^+^ (calc. for C_21_H_27_N_2_O_10_, 467.1660).

**Ginkgoside B (2)**. Yellow-brownish solid; [α]_D_^25^ + 14.2 (c 0.1, MeOH); IR (KBr) 3423, 2951, 1721, 1261 cm^–1^; ^1^H and ^13^C NMR data: see Table 1. HRESIMS *m/z* 453.1510 [M + H]^+^ (calc. for C_20_H_25_N_2_O_10_, 453.1504).

### Concise synthesis of compounds **1** and **2**

Ethyl bromide (0.95 ml, 12.6 mmol, 1.1 equiv.) and K_2_CO_3_ (1.90 g, 13.7 mmol, 1.2 equiv.) were added to a solution of indoleacetic acid (**3**) (2.0 g, 11.4 mmol, 1.0 equiv.) in DMF (20 ml), and the mixture was stirred at 130 °C for 2 hours. After cooling to room temperature, the reaction mixture was diluted with water and extracted with ethyl acetate for three times. The extracts were evaporated to dryness, then subjected to CC (petroleum ether:ethyl acetate 10:1-5:1) to give **4** (2.18 g, 94%) as a dark brown liquid. ^1^H NMR (600 MHz, CDCl_3_): *δ*_H_ 8.22 (1 H, brs, *N*-H), 7.60 (1 H, d, *J* = 7.8 Hz), 7.23 (1 H, d, *J* = 8.1 Hz), 7.16 (1 H, t, *J* = 7.5 Hz), 7.11 (1 H, t, *J* = 7.2 Hz), 6.97 (1 H, s), 4.15 (2 H, q, *J* = 7.1 Hz), 3.74 (2 H, s), 1.24 (1 H, t, *J* = 7.1 Hz). ^13^C NMR (150 MHz, CDCl_3_): *δ*_C_ 172.4, 136.2, 126.8, 123.1, 122.0, 119.1, 118.3, 110.7, 108.1, 60.8, 31.4, 14.1. HRESIMS *m/z* 204.1025 [M + H]^+^ (calcd for C_12_H_14_NO_2_ 204.1023).

Compound **4** (1.5 g, 7.4 mmol, 1.0 equiv.) was solved in HCl/EtOH (1:1, 20 ml) and then cooled to 0 °C. Pyridine borane (3.8 ml, 36.9 mmol, 5.0 equiv.) was added to the above solution slowly under an atmosphere of nitrogen. The mixture was stirred at 0 °C for 20 min. When the reaction finished, the mixture was concentrated under reduced pressure and a solution of Na_2_CO_3_ was added to adjust to pH 8. The resultant mixture was extracted with ethyl acetate for three times. The extracts were then washed (saturated sodium chloride solution), dried (magnesium sulphate) and evaporated to give **5** (1.44 g, 95%) as a dark brown liquid. ^1^H NMR (600 MHz, CDCl_3_): *δ*_H_ 7.01 (1 H, d, *J* = 7.3 Hz), 6.97 (1 H, t, *J* = 7.6 Hz), 6.64 (1 H, t, *J* = 7.4 Hz), 6.57 (1 H, d, *J* = 7.8 Hz), 4.10 (2 H, q, *J* = 7.1 Hz), 3.71 (1 H, t, *J* = 8.8 Hz), 3.65 (1 H, dt, *J* = 21.0, 7.4 Hz), 3.19 (1 H, dd, *J* = 8.9, 6.7 Hz), 2.69(1 H, dd, *J* = 16.0, 5.4 Hz), 2.48 (1 H, dd, *J* = 16.0, 9.1 Hz), 1.20 (1 H, t, *J* = 7.1 Hz). ^13^C NMR (150 MHz, CDCl_3_): *δ*_C_ 172.5, 151.2, 131.1, 127.8, 123.7, 118.9, 109.9, 60.6, 53.2, 39.0, 38.3, 14.7. HRESIMS *m/z* 206.1181 [M + H]^+^ (calcd for C_12_H_16_NO_2_ 206.1179).

A mixture of **5** (0.6 g, 2.93 mmol, 1.0 equiv.), D-glucose (0.58 g, 3.21 mmol, 1.1 equiv.) and ethanol (20 mL) was heated to reflux for 12 hours. The above mixture was concentrated and subjected to CC (CH_2_Cl_2_:MeOH 10:1-5:1) to give **6** (dark brown solid, 666 mg, 62%) as an inseparable mixture of isomers in 1:1 ratio. ^1^H NMR (600 MHz, CD_3_OD): *δ*_H_ 7.02 (4 H, m), 6.64 (4 H, m), 4.78 (2 H, d, *J* = 8.9 Hz), 4.16 (2 H, m), 4.12 (2 H, m), 3.88 (1 H, t, *J* = 8.8 Hz), 3.80 (1 H, m), 3.77 (2 H, m), 3.67 (1 H, m), 3.60 (3 H, m), 3.52 (2 H, m), 3.46 (2 H, m), 3.40 (1 H, dd, *J* = 9.1, 2.7 Hz), 3.29 (3 H, m), 2.89 (1 H, dd, *J* = 16.2, 5.2 Hz), 2.58 (2 H, m), 2.50 (1 H, dd, *J* = 16.2, 9.3 Hz), 1.25 (3 H, t, *J* = 7.1 Hz),1.21 (3 H, t, *J* = 7.1 Hz); ^13^C NMR (150 MHz, CD_3_OD): *δ*_C_ 174.3, 174.1, 151.6, 151.2, 133.8, 133.4, 129.0, 128.8, 125.3, 124.4, 120.0, 119.9, 109.4, 109.3, 86.9, 86.7, 79.3, 79.2, 72.1, 71.6, 62.7, 61.7, 53.3, 52.5, 39.6, 40.9, 38.1, 38.0 14.6, 14.5. HRESIMS *m/z* 368.1709 [M + H]^+^ (calcd for C_18_H_26_NO_7_ 368.1714).

To a solution of **6** (0.65 g, 1.77 mmol, 1.0 equiv.) in ethanol (20 mL), 2,3-dichloro-5,6-dicyano-1,4-benzoquinone (DDQ, 485 mg, 2.12 mmol, 1.2 equiv.) was added slowly at room temperature. After 30 minutes, the mixture was filtered and the filtrate was evaporated to dryness. The concentrates were resolved in water and extracted with ethyl acetate for three times. Compound **7** (dark brown solid, 246 mg) was obtained in 38% yield by chromatography on a silica column using CH_2_Cl_2_:MeOH (20:1-10:1) as the eluent. ^1^H NMR (600 MHz, CD_3_OD): *δ*_H_ 7.54 (2 H, t, *J* = 8.7 Hz), 7.38 (1 H, s), 7.20 (1 H, t, *J* = 8.1 Hz), 7.10 (1 H, t, *J* = 7.8 Hz), 5.45 (1 H, d, *J* = 9.0 Hz), 4.17 (2 H, q, *J* = 7.1 Hz), 3.91 (2 H, dt, *J* = 12.2, 5.6 Hz), 3.78 (2 H, s), 3.72 (1 H, dd, *J* = 12.2, 5.7 Hz), 3.60 (2 H, m), 3.52 (1 H, m), 1.27 (3 H, t, *J* = 7.1 Hz). ^13^C NMR (150 MHz, CD_3_OD): *δ*_C_ 174.1, 138.3, 129.5, 125.6, 123.0, 120.8, 120.0, 111.5, 110.0, 87.0, 80.5, 78.7, 73.7, 71.1, 62.7, 61.7, 31.8, 14.6. HRESIMS *m/z* 366.1553 [M + H]^+^ (calcd for C_18_H_24_NO_7_ 366.1548).

A mixture of **7** (0.20 g, 0.55 mmol, 1.0 equiv.), sodium hydroxide (300 mg), ethanol:water (1:1, 8 mL) was stirred at reflux for 1 hour. The mixture was concentrated and 2 M HCl was added to adjust to pH 2. The resultant was concentrated under reduced pressure and purified by column chromatography (CH_2_Cl_2_:EtOH:HOAC 10:1:0.01 to 4:1:0.01) to afford 175 mg (95% yield) of compound **8**. ^1^H NMR (600 MHz, CD_3_OD): *δ*_H_ 7.57 (1 H, d, *J* = 7.7 Hz), 7.53 (1 H, d, *J* = 8.3 Hz), 7.35 (s, 1 H), 7.18 (1 H, t, *J* = 7.6 Hz), 7.08 (1 H, t, *J* = 7.3 Hz), 5.44 (1 H, d, *J* = 9.0 Hz,), 3.99–3.86 (2 H, m), 3.79–3.70 (3 H, m), 3.66–3.59 (2 H, m), 3.56–3.49 (m, 1 H). HRESIMS *m/z* 338.1240 [M + H]^+^ (calcd for C_16_H_20_NO_7_ 338.1231).

A mixture of **8** (150 mg, 0.45 mmol, 1.0 equiv.), L-glutamic acid methyl ester or L-aspartic acid methyl ester hydrochloride (0.67 mmol, 1.5 equiv.), EDC (129 mg, 0.67 mmol, 1.5 equiv.), and NMM (92 *µ*L, 0.67 mmol, 1.5 equiv.) in THF was stirred at room temperature for 16 hours. After filtration, the filtrate was washed with water and then subjected to to CC (CH_2_Cl_2_:MeOH 20:1-10:1) to afford **9** (187 mg, 85% yield, white solid) and **10** (171 mg, 80% yield, white solid). ^1^H NMR of **9** (600 MHz, CD_3_OD): *δ*_H_ 7.56 (2 H, d, *J* = 8.9 Hz), 7.39 (1 H, s), 7.21 (1 H, t, *J* = 8.1 Hz), 7.11 (1 H, t, *J* = 7.8 Hz), 5.46 (1 H, d, *J* = 9.0 Hz), 4.49 (1 H, dd, *J* = 9.3, 5.2 Hz), 3.94 (1 H, m), 3.90 (1 H, dd, *J* = 12.2, 2.2 Hz), 3.76–3.71 (3 H, m), 3.71 (3 H, s), 3.66 (3 H, s), 3.62 (1 H, d, *J* = 4.6 Hz), 3.59 (1 H, dd, *J* = 5.7, 2.3 Hz), 3.52 (1 H, m), 2.40 (2 H, t, *J* = 7.4 Hz), 2.18 (1 H, m), 1.95 (1 H, m). ^13^C NMR of **9** (150 MHz, CD_3_OD): *δ*_C_ 174.8, 174.7, 173.6, 138.0, 129.6, 125.5, 123.0, 121.1, 119.7, 111.5, 110.5, 86.2, 80.3, 78.8, 73.0, 70.8, 62.3, 54.5, 52.7, 52.2, 33.3, 31.0, 27.6. HRESIMS *m/z* 495.1974 [M + H]^+^ (calcd for C_23_H_30_N_2_O_10_ 495.1979).

^1^H NMR of **10** (600 MHz, CD_3_OD): *δ*_H_ 7.54 (1 H, d, *J* = 8.3 Hz), 7.51 (1 H, d, *J* = 7.9 Hz), 7.37 (1 H, s), 7.19 (1 H, t, *J* = 7.7 Hz), 7.09 (1 H, t, *J* = 7.2 Hz), 5.44 (1 H, d, *J* = 9.0 Hz), 4.79 (1 H, dd, *J* = 7.1, 5.4 Hz), 3.93–3.87 (2 H, m), 3.73–3.69 (3 H, m), 3.68 (3 H, s), 3.61 (1 H, m), 3.59 (3 H, s), 3.57 (1 H, m), 3.51 (1 H, m), 2.54 (2 H, ddd, *J* = 23.7, 16.6, 6.2). ^13^C NMR of **10** (150 MHz, CD_3_OD): *δ*_C_ 174.5, 172.7, 172.6, 138.9, 129.7, 126.0, 123.1, 121.2, 120.5, 111.3, 110.2, 86.3, 80.8, 79.4, 74.2, 70.9, 62.4, 53.0, 52.5, 50.7, 36.9, 33.6. HRESIMS *m/z* 481.1825 [M + H]^+^ (calcd for C_22_H_28_N_2_O_10_ 481.1822).

A mixture of **9** or **10** (0.31 mmol, 1.0 equiv.), sodium hydroxide (480 mg), ethanol:water (1:1, 6 mL) was stirred at reflux for 1 hour. The mixture was concentrated and 2 M HCl was added to adjust to pH 2. The extract was concentrated and purified by CC (CH_2_Cl_2_:EtOH 10:1-2:1) to give **11** [136 mg, [α]_D_^25^ + 7.8 (c 0.1, MeOH)] and **12** [133 mg, [α]_D_^25^ + 6.5 (c 0.1, MeOH)] in 94% yield. ^1^H NMR of **11** (600 MHz, CD_3_OD): *δ*_H_ 7.54 (2 H, d, *J* = 8.0 Hz), 7.37 (1 H, s), 7.18 (1 H, m), 7.09 (1 H, m), 5.44 (1 H, d, *J* = 8.7 Hz), 4.47 (1 H, m), 3.96-3.87 (2 H, m), 3.76–3.61 (3 H, m), 3.60–3.52 (3 H, m), 2.36 (2 H, brs), 2.18 (1 H, m), 1.93 (1 H, m). ^13^C NMR of **11** (150 MHz, CD_3_OD): *δ*_C_ 176.6, 176.2, 174.8, 138.5, 130.0, 126.9, 123.1, 121.2, 119.5, 111.8, 110.3, 86.9, 80.3, 78.5, 73.4, 70.8, 62.8, 53.4, 33.5, 31.0, 27.7. HRESIMS *m/z* 467.1666 [M + H]^+^ (calcd for C_21_H_26_N_2_O_10_ 467.1659).

^1^H NMR of **12** (600 MHz, CD_3_OD): *δ*_H_ 7.56 (2 H, d, *J* = 8.0 Hz), 7.41 (1 H, s), 7.21 (1 H, t, *J* = 7.6 Hz), 7.11 (1 H, t, *J* = 7.1 Hz), 5.48 (1 H, d, *J* = 9.0 Hz), 4.78 (1 H, m), 3.96–3.90 (2 H, m), 3.78–3.71 (3 H, m), 3.66–3.60 (2 H, m), 3.54 (1 H, m), 2.85 (2 H, qd, *J* = 16.8, 5.7 Hz). ^13^C NMR of **12** (150 MHz, CD_3_OD): *δ*_C_ 174.4, 174.3, 174.2, 138.5, 129.6, 126.7, 123.2, 121.0, 119.8, 111.4, 110.5, 86.5, 80.3, 78.5, 73.7, 70.8, 62.0, 49.9, 36.4, 34.3. HRESIMS *m/z* 453.1509 [M + H]^+^ (calcd for C_20_H_24_N_2_O_10_ 453.1495).

### Cytotoxic activity assay

The human cancer cell lines (HepG2, MCF-7, HT-29 and A-549) were purchased from School of Basic Medicine of Peking Union Medical College (Beijing, China). The cells were cultured in DMEM or RPMI-1640 medium (Corning, USA), supplemented with 10% fetal bovine serum (FBS) (Gibco, USA) and antibiotics (100 units/mL penicillin and streptomycin) (Hyclone, USA) at 37 °C in a humidified atmosphere of 5% CO_2_. Cell viability was assessed using the 3-(4,5-dimethylthiazol-2-yl)-2,5-diphenyltetrazolium bromide (MTT) (Sigma, MO) assay^20^. The cells were seeded into 96-well cell culture plate at a density of 1 × 10^4^ cell/well and incubated at 37 °C for 24 h. And then Cells were treated with various concentrations of compounds **1** or **2**. After 48 h incubation, 10 *μ*l MTT (5 mg/ml in PBS) solution was added into each well. The plates were incubated for another 4 h at 37 °C. After removal of the medium, the cells were lysed by 100 *μ*l DMSO in 10 min. Absorbance values were determined at 450 nm using a microplate reader (Multiskan FC, Thermo, USA).

### *In vitro* anti-inflammation assessment

The murine RAW264.7 cell lines were purchased from School of Basic Medicine of Peking Union Medical College (Beijing, China). The cells were culture in DMEM medium, supplemented with 10% FBS and antibiotics at 37 °C in a humidified atmosphere of 5% CO_2_. To evaluate the anti-inflammation effects of compounds **1** and **2**, cells divided into six groups: control; LPS treated; LPS + compound **1**; LPS + compound **2** treated; compound **1** treated; compound **2** treated. Each group had three replicated wells. Cells were seeded into 6-well cell culture plate at a density of 2 × 10^5^ cell/well and incubated at 37 °C. After 24 h, cells were subjected to compound **1** or **2** at the concentration of 50 *μ*M for another 24 h. Cells then treated with LPS (500 ng/ml) (Sigma, MO) for 12 h. Subsequently, cells were harvested and extracted for analysis. The quantifification of mRNA in macrophage cells was performed using a previously standardized method^21,22^.

## Supplementary information

Supplementary information
