# Supplementary material for: Isolation of two rare N-glycosides from Ginkgo biloba and their anti-inflammatory activities
Source: Sci Rep. 2020 Apr 7;10:5994. doi: 10.1038/s41598-020-62884-1 (PMC7138816; doi:10.1038/s41598-020-62884-1)
Supplement: Supplementary file 1 — Supplementary information [file 41598_2020_62884_MOESM1_ESM.pdf]

Supporting Information

Isolation of two rare *N*-glycosides from *Ginkgo biloba* and their anti-inflammatory activities

Jin-Tang Cheng,[‡a] Cong Guo,[‡a] Wen-Jin Cui,[‡a] Qing Zhang,[a] Shu-Hui Wang,[a] Qing-He Zhao,[a] De-Wen Liu,[a] Jun Zhang,[a] Sha Chen,[a] Chang Chen,[a] Yan Liu,[a] Zheng-Hong Pan[b] and An Liu[*a]

[a] Institute of Chinese Materia Medica, China Academy of Chinese Medical Sciences, Beijing 100700, China

[b] Guangxi Key Laboratory of Functional Phytochemicals Research and Utilization, Guangxi Institute of Botany, Guangxi Zhuang Autonomous Region and Chinese Academy of Sciences, Guilin 541006, China

‡ J.-T. Cheng, C. Guo and W.-J. Cui contributed equally.

* Corresponding author. Tel.: +86-10-6401-4411 (ext. 2848); Fax: +86-10-6401-3996; E-mail address: la62@163.com (A. Liu)

Contents of Supporting Information

Figure S1. ^{1}H NMR spectrum of compound **1**......4

Figure S2. DEPT spectra of compound **1**......5

Figure S3. ^{1}H-^{1}H COSY spectrum of compound **1**......6

Figure S4. HSQC spectrum of compound **1**......7

Figure S5. HMBC spectrum of compound **1**......8

Figure S6. ROESY spectrum of compound **1**...... 9

Figure S7. HRESIMS of compound **1**......10

Figure S8. ^{1}H NMR spectrum of compound **2**...... 11

Figure S9. ^{13}C NMR spectrum of compound **2**......12

Figure S10. HSQC spectrum of compound **2**......13

Figure S11. ^{1}H-^{1}H COSY spectrum of compound **2**...... 14

Figure S12. HMBC spectrum of compound **2**...... 15

Figure S13. HRESIMS of compound **2**......16

Figure S14. ^{1}H NMR spectrum of compound **4**......17

Figure S15. ^{13}C NMR spectrum of compound **4**......18

Figure S16. ^{1}H NMR spectrum of compound **5**......19

Figure S17. ^{13}C NMR spectrum of compound **5**......20

Figure S18. ^{1}H NMR spectrum of compound **6**......21

Figure S19. ^{13}C NMR spectrum of compound **6**......22

Figure S20. ^{1}H NMR spectrum of compound **7**......23

Figure S21. ^{13}C NMR spectrum of compound **7**......................................24

Figure S22. ^{1}H NMR spectrum of compound **8**.......................................25

Figure S23. ^{1}H NMR spectrum of compound **9**.......................................26

Figure S24. ^{13}C NMR spectrum of compound **9**......................................27

Figure S25. ^{1}H NMR spectrum of compound **10**......................................28

Figure S26. ^{13}C NMR spectrum of compound **10**....................................29

Figure S27. ^{1}H NMR spectrum of compound **11**......................................30

Figure S28. ^{13}C NMR spectrum of compound **11**....................................31

Figure S29. ^{1}H NMR spectrum of compound **12**......................................32

Figure S30. ^{13}C NMR spectrum of compound **12**....................................33

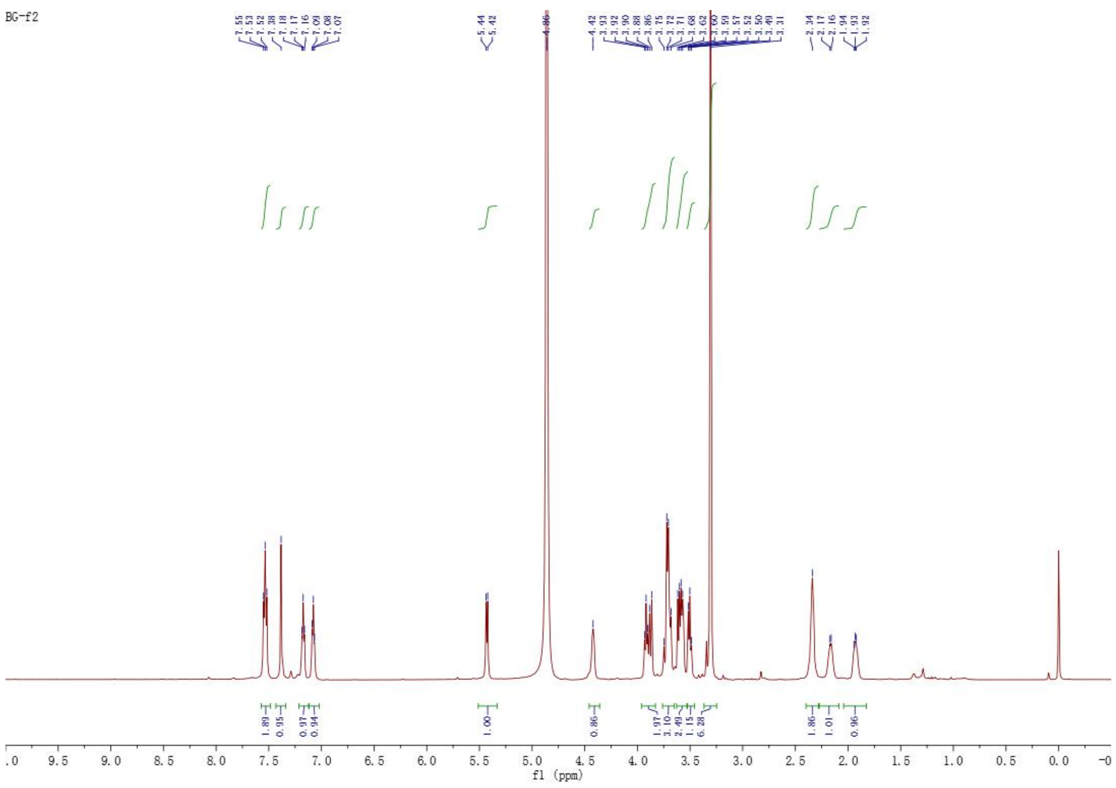

Figure S1. ^{1}H NMR spectrum of compound 1

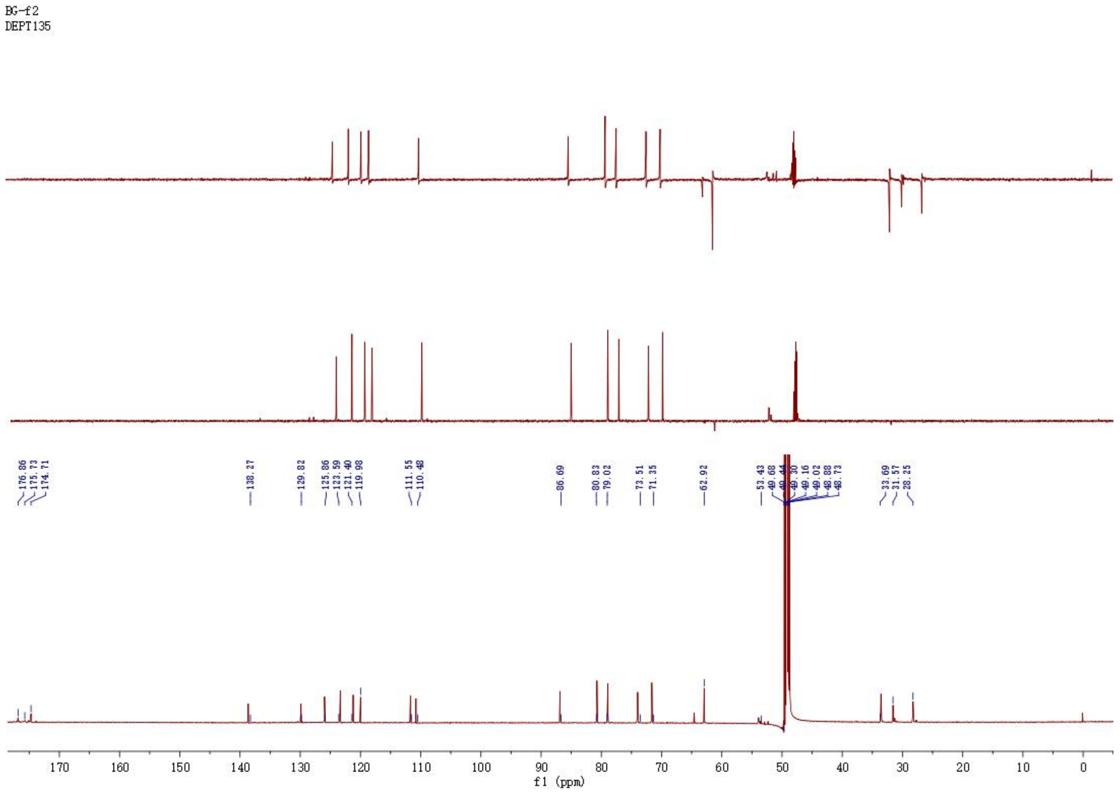

Figure S2. DEPT spectra of compound 1

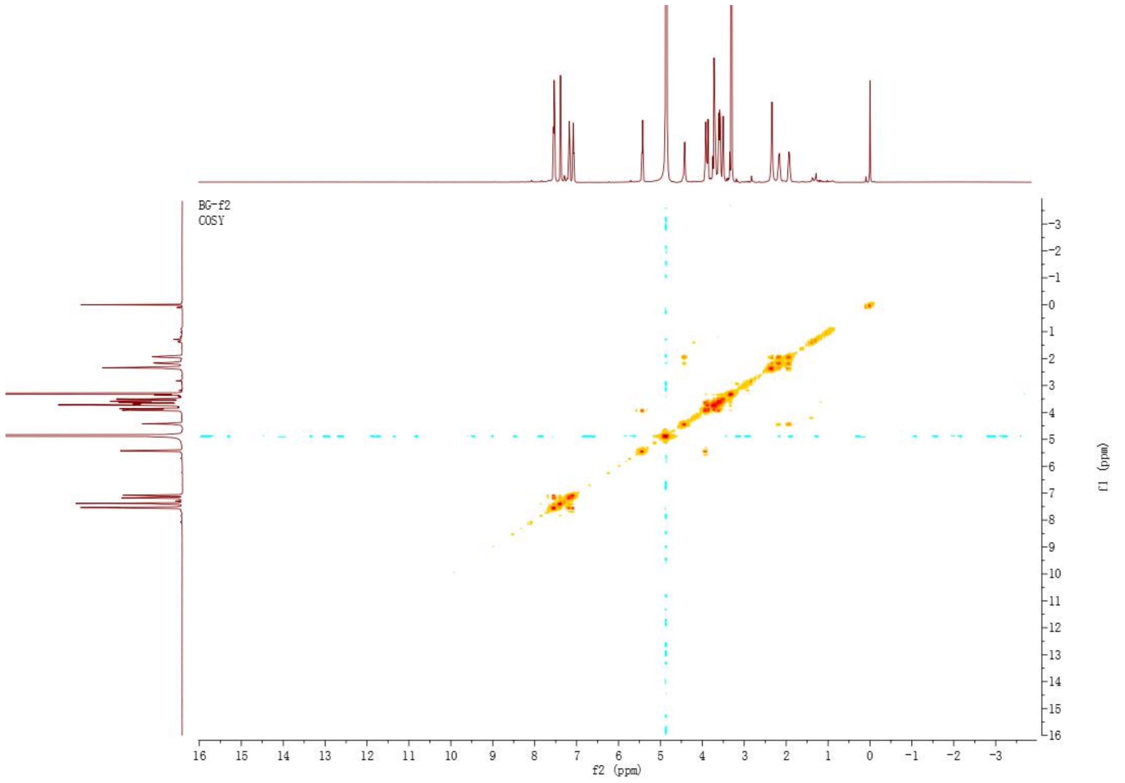

Figure S3. ^{1}H-^{1}H COSY spectrum of compound 1

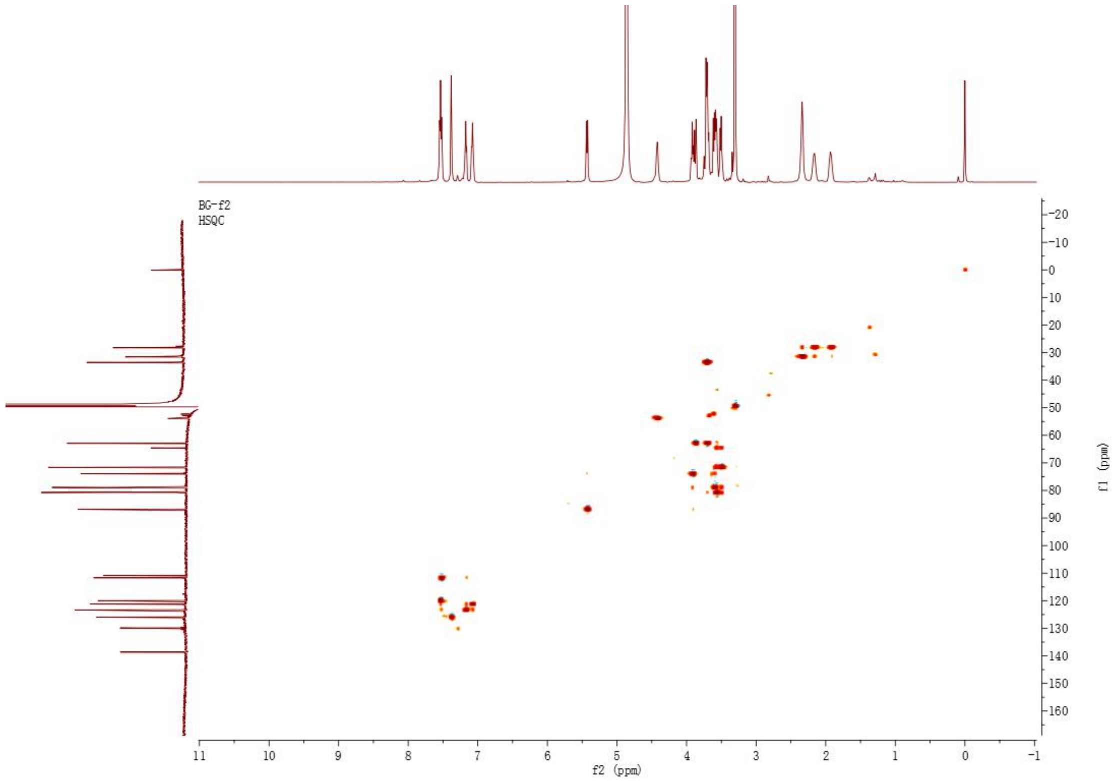

Figure S4. HSQC spectrum of compound 1

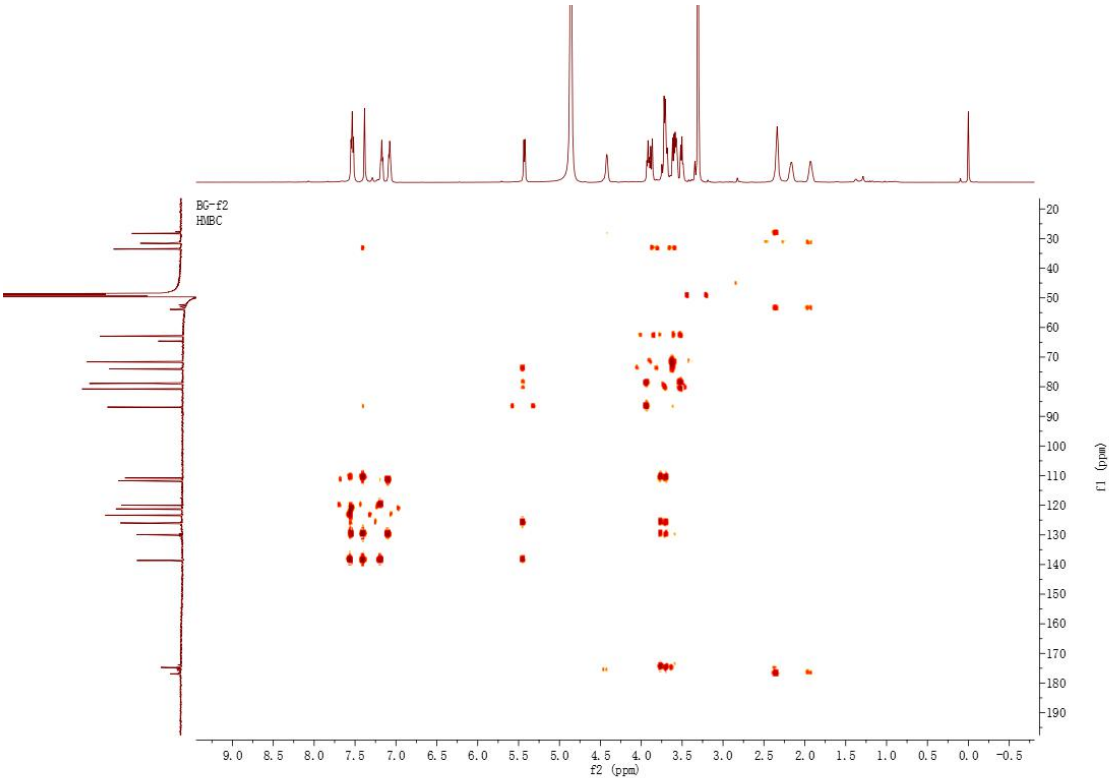

Figure S5. HMBC spectrum of compound 1

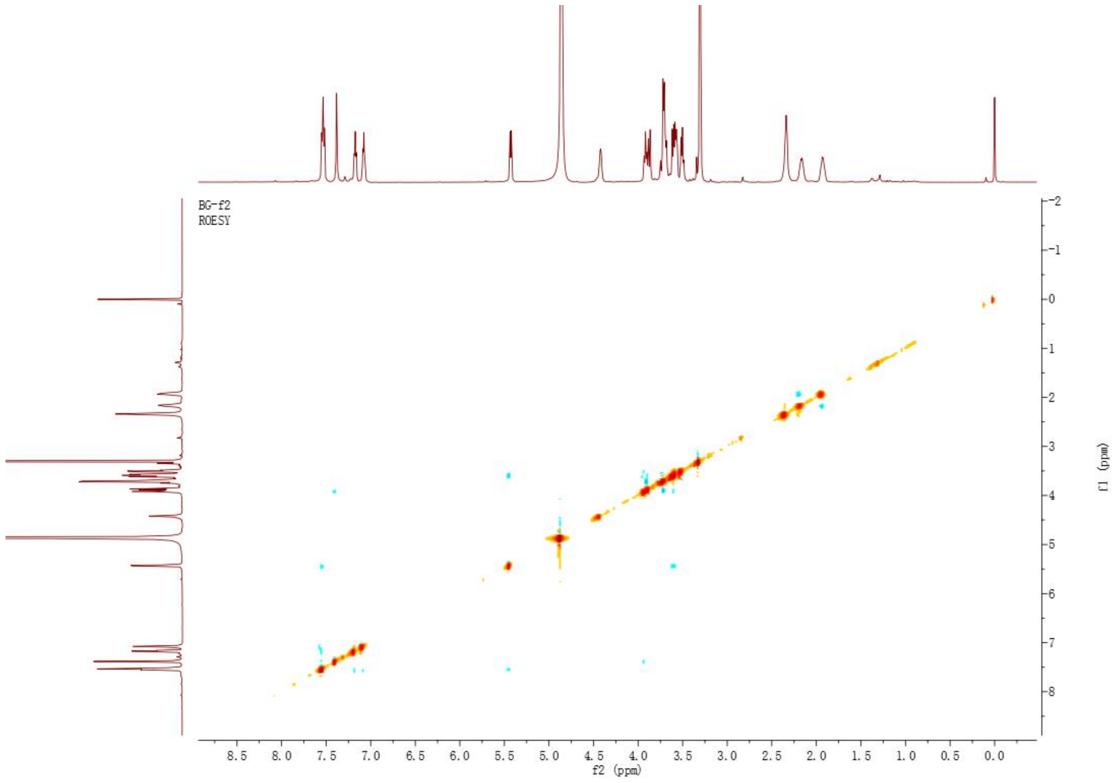

Figure S6. ROESY spectrum of compound 1

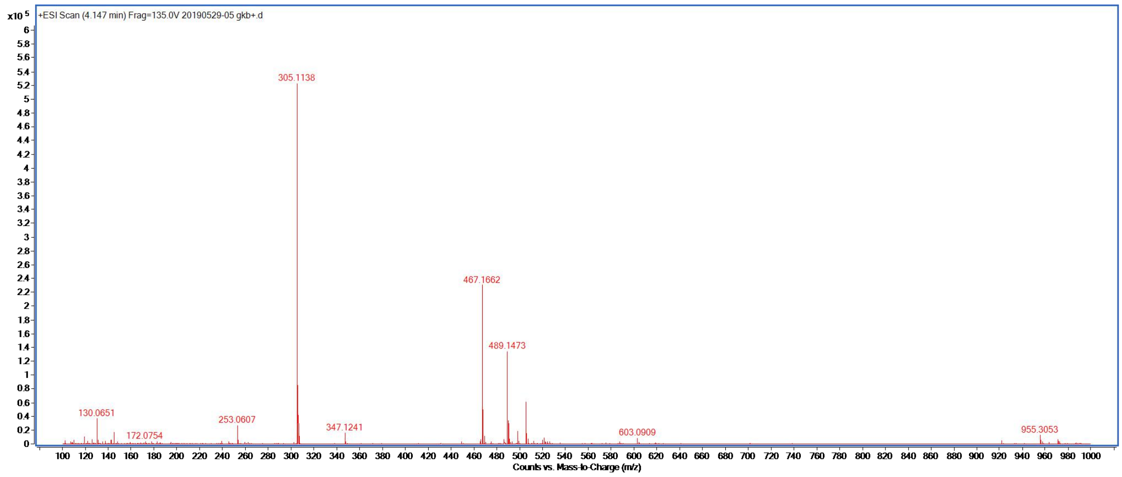

Figure S7. HRESIMS of compound 1

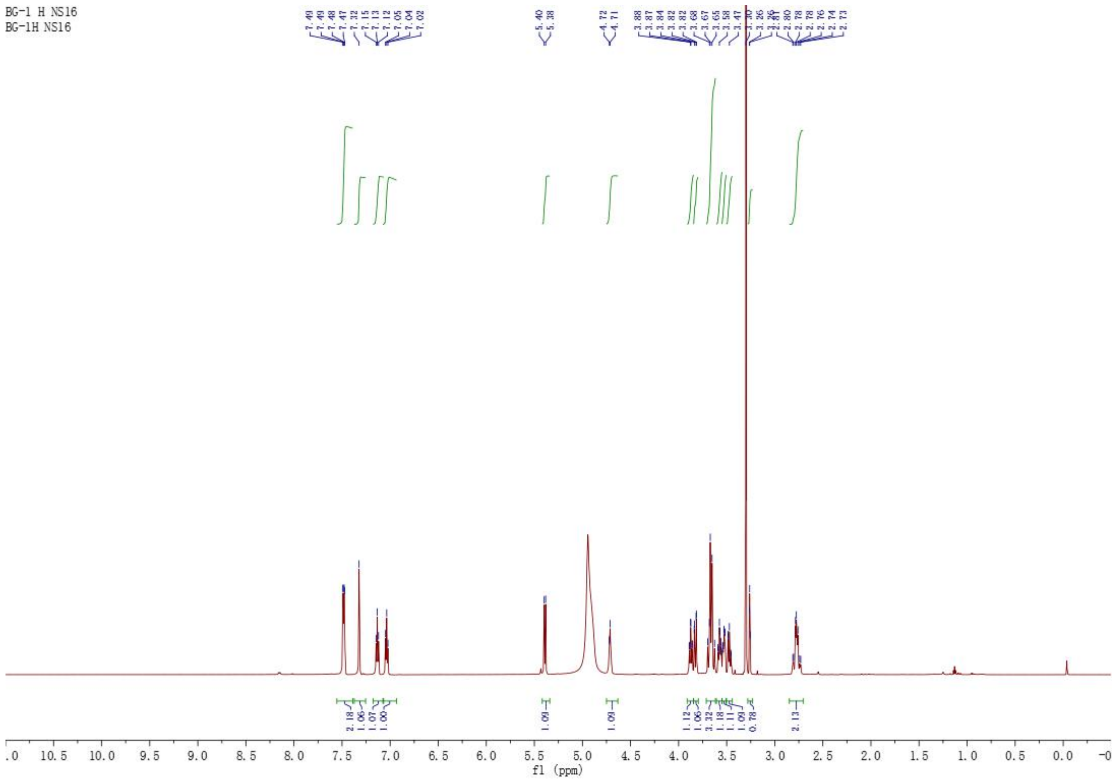

Figure S8. ^{1}H NMR spectrum of compound 2

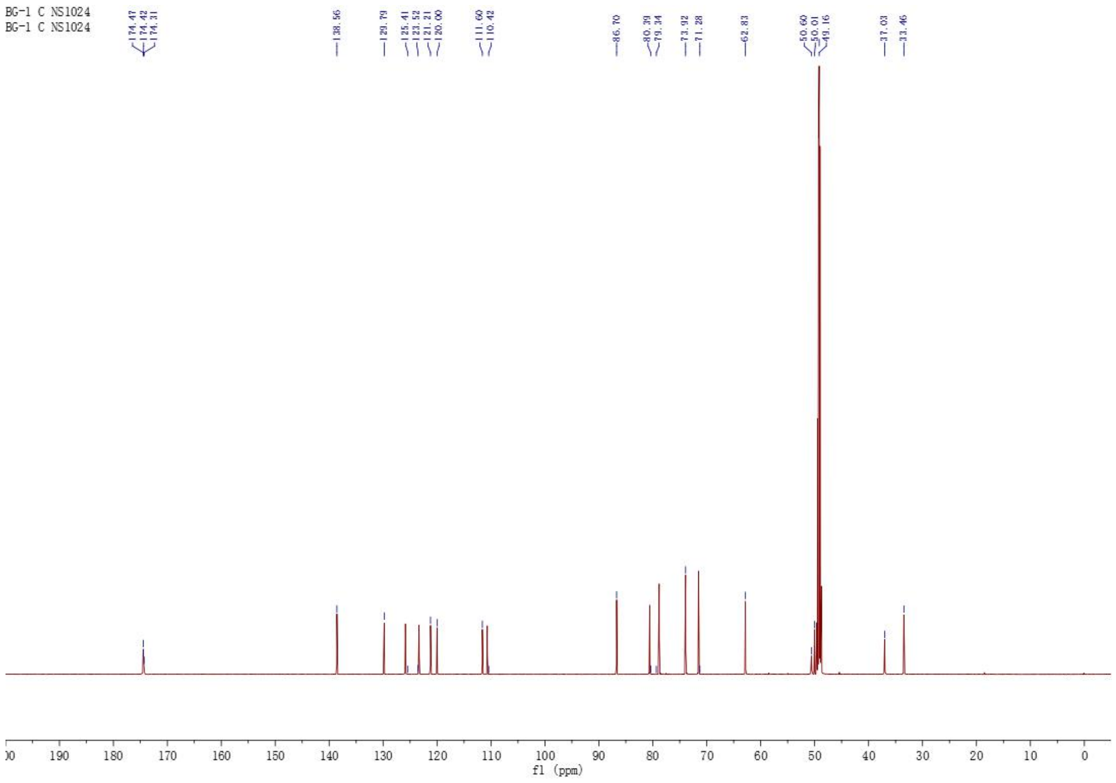

Figure S9. ^{13}C NMR spectrum of compound 2

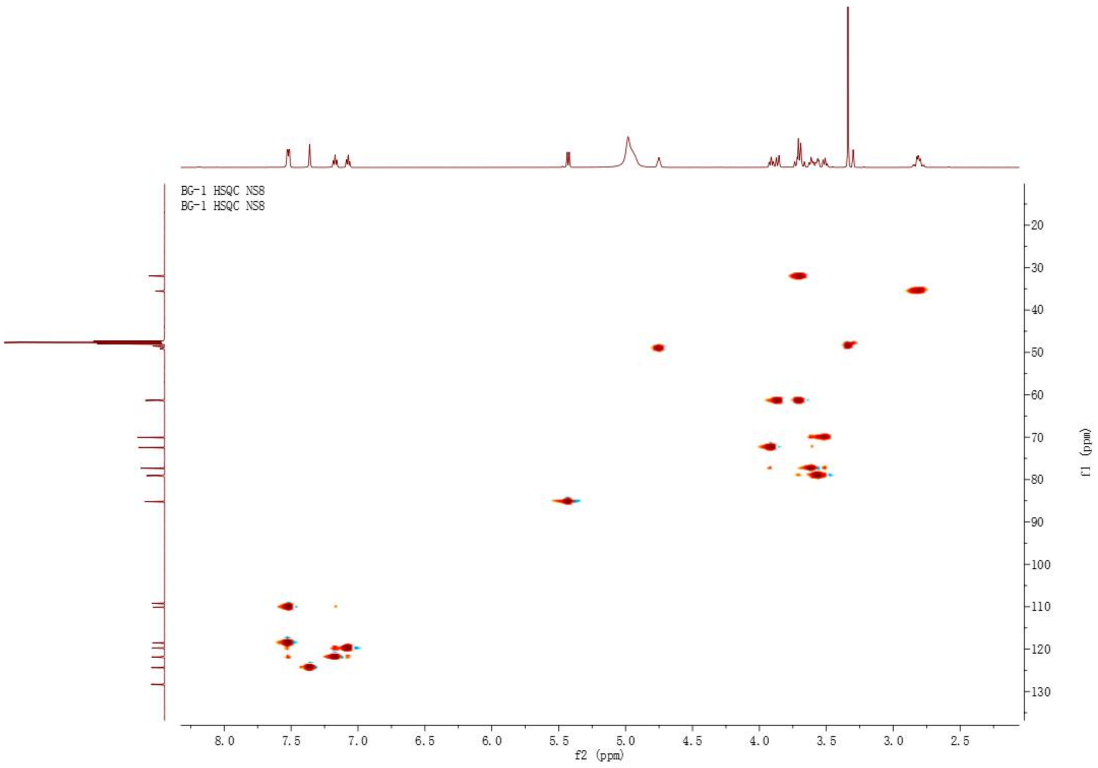

Figure S10. HSQC spectrum of compound 2

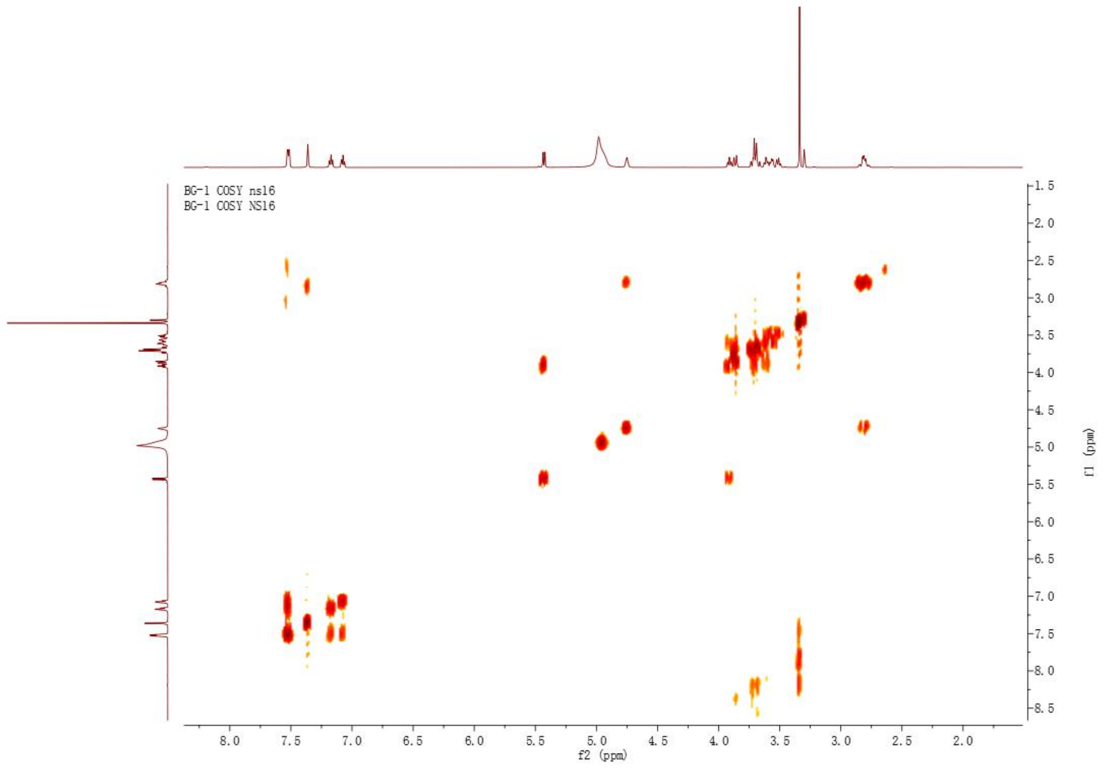

Figure S11. ^{1}H-^{1}H COSY spectrum of compound 2

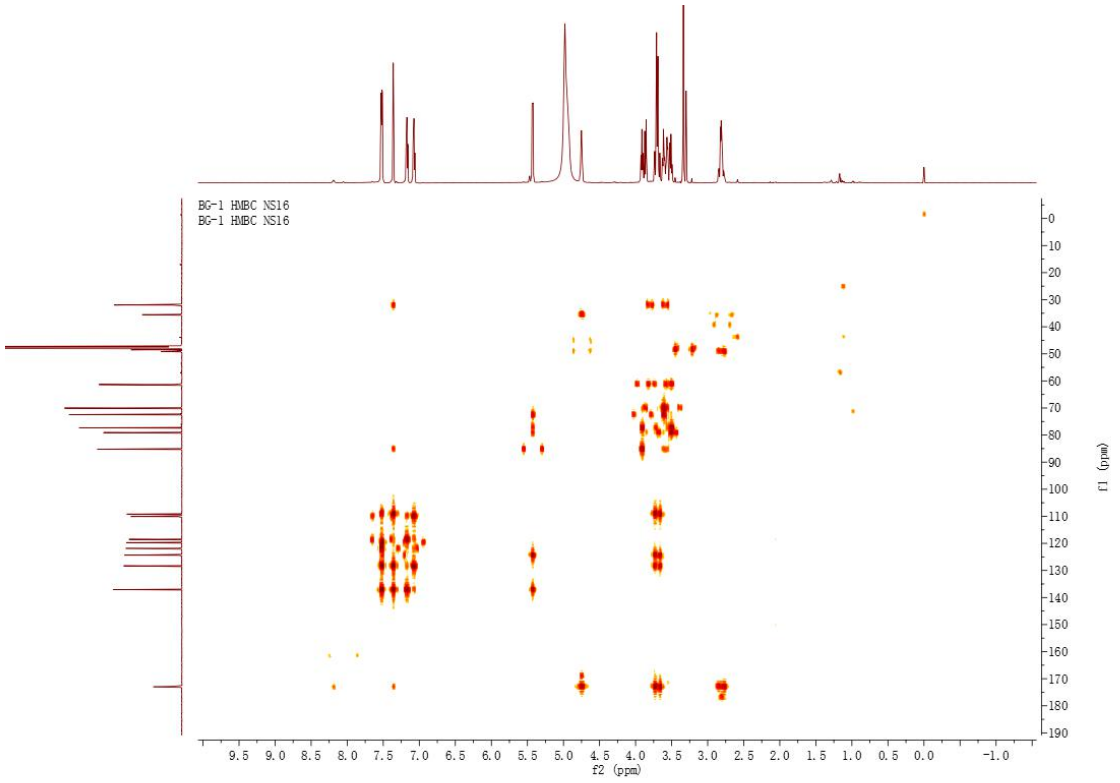

Figure S12. HMBC spectrum of compound 2

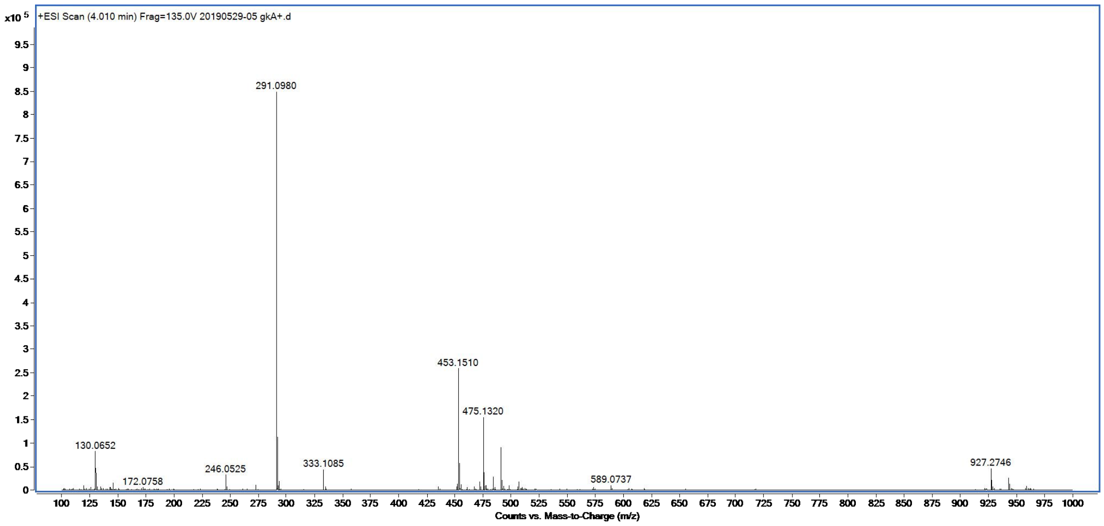

Figure S13. HRESIMS of compound 2

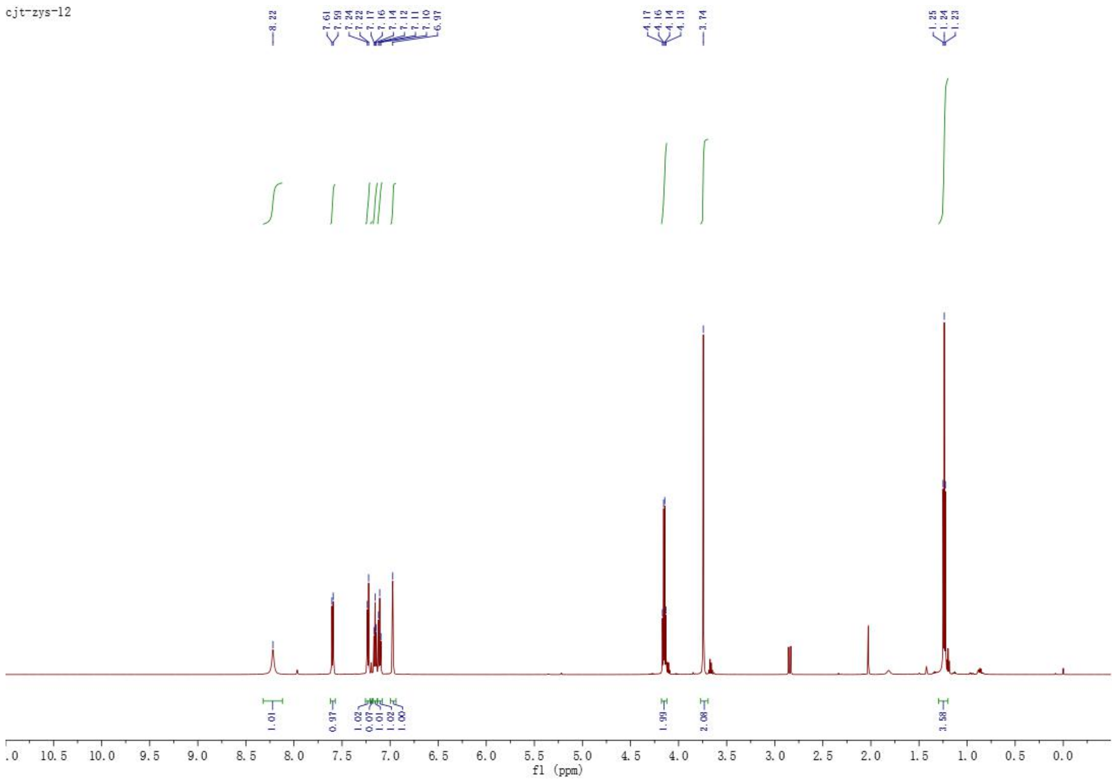

Figure S14. **^{1}H NMR spectrum of compound 4**

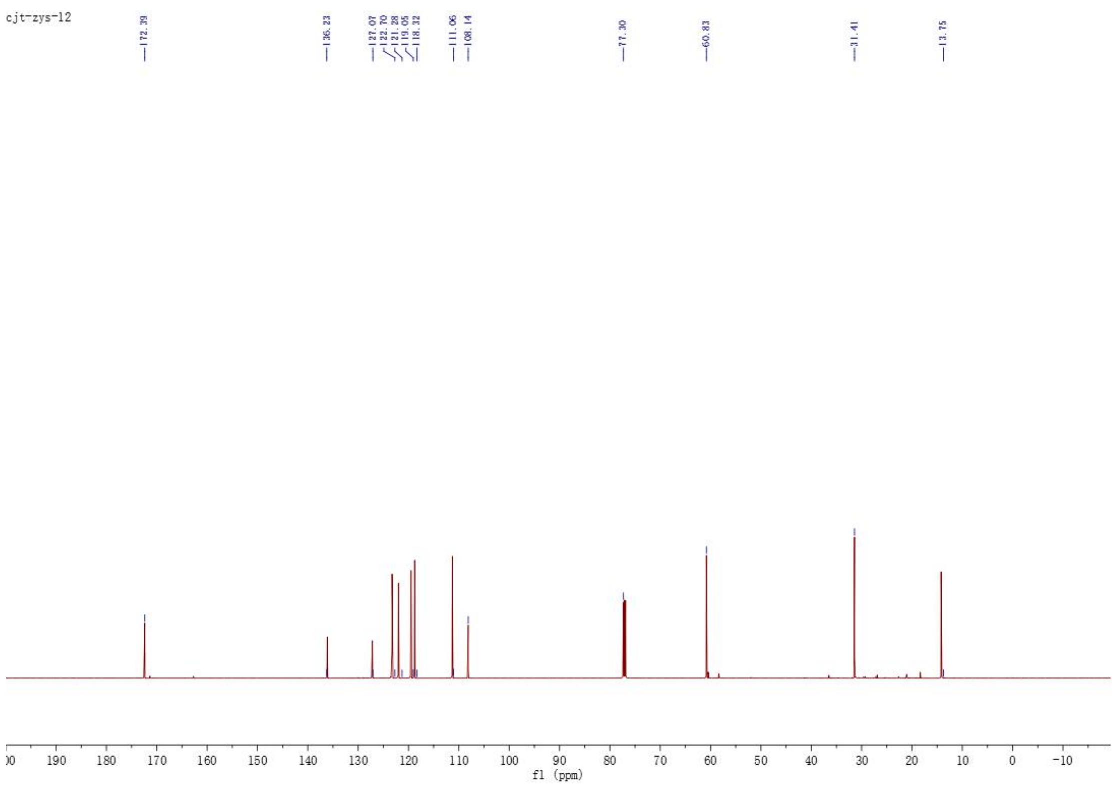

Figure S15. ^{13}C NMR spectrum of compound 4

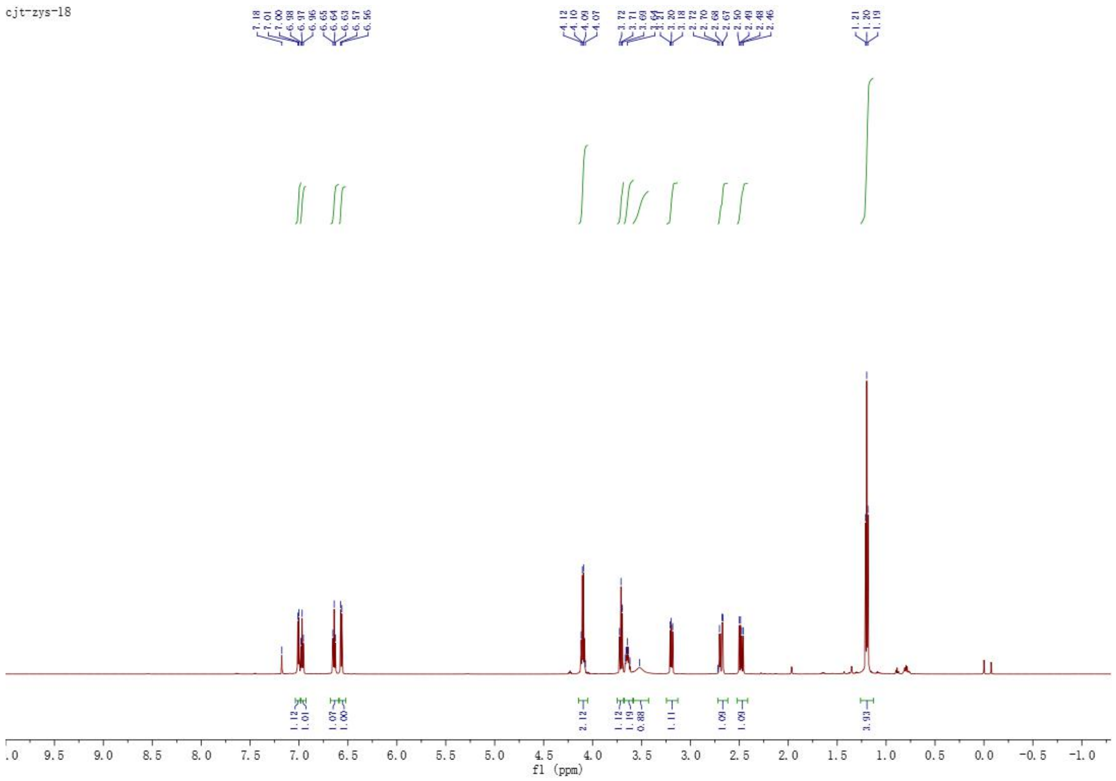

Figure S16. ^{1}H NMR spectrum of compound 5

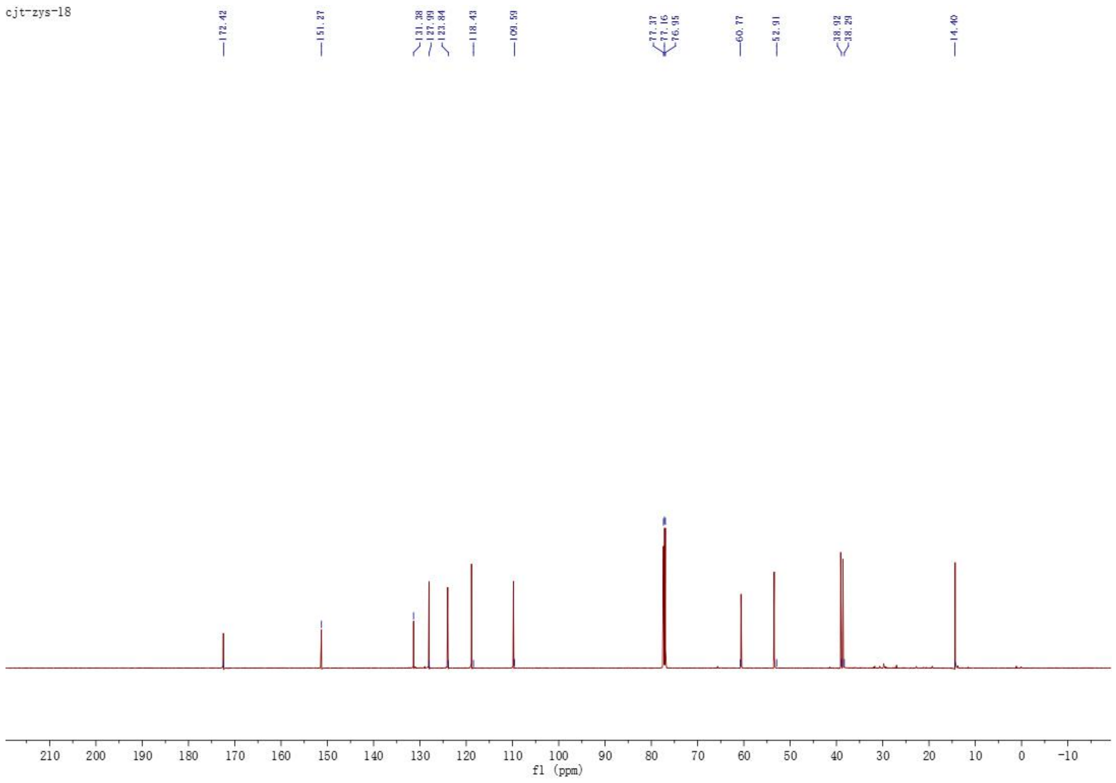

Figure S17. ^{13}C NMR spectrum of compound 5

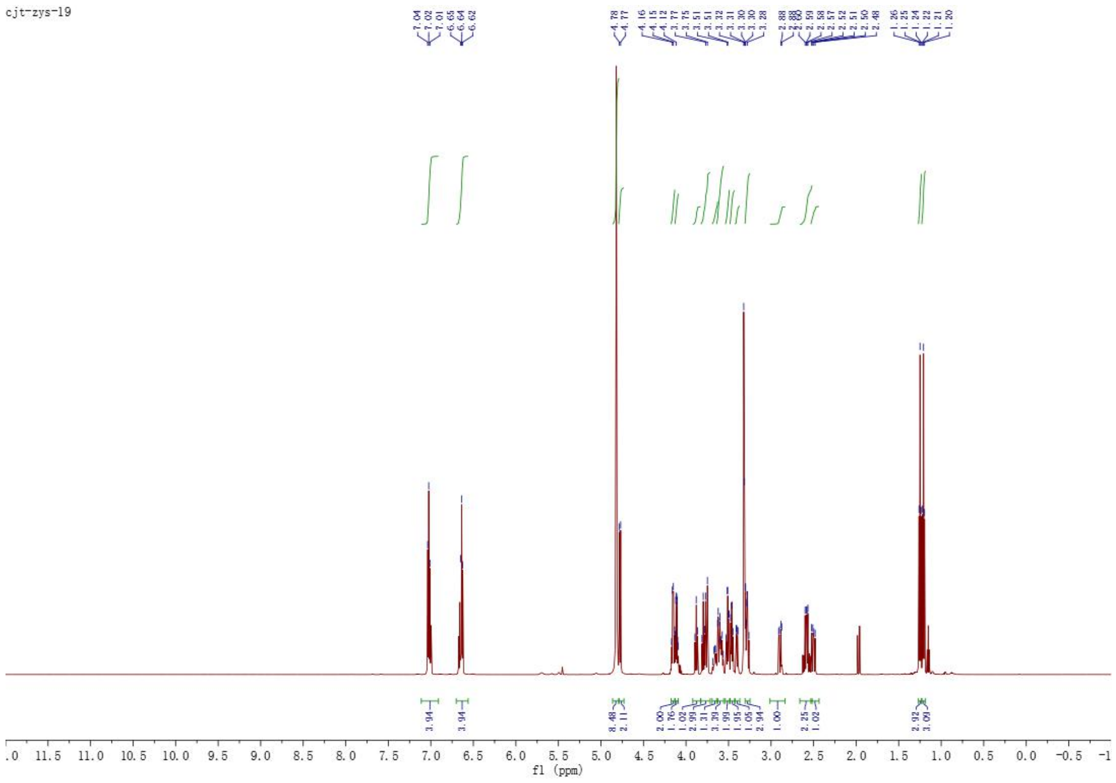

Figure S18. ^{1}H NMR spectrum of compound 6

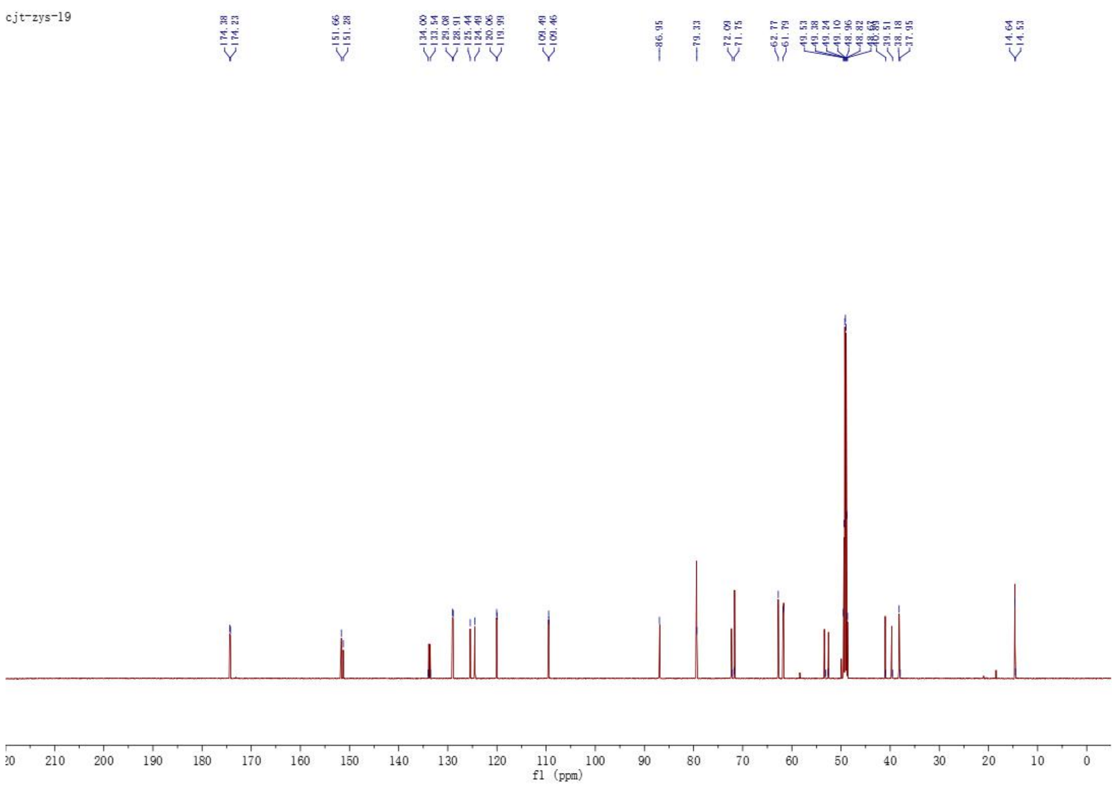

Figure S19. ^{13}C NMR spectrum of compound 6

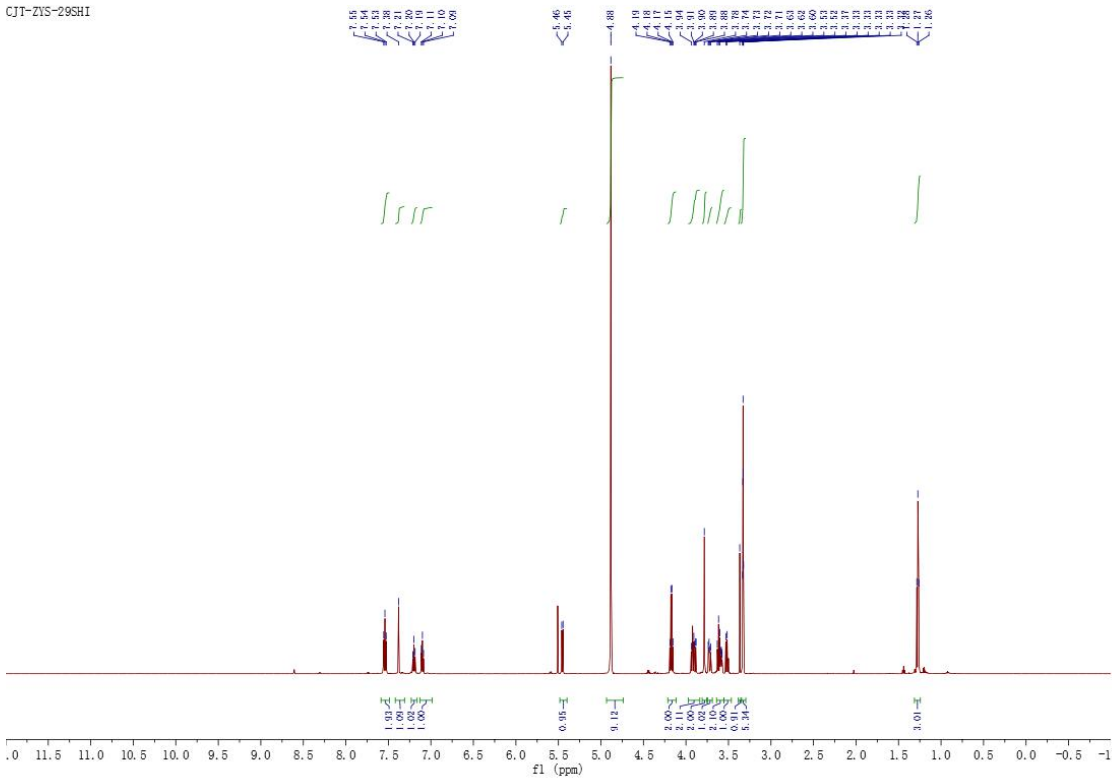

Figure S20. ^{1}H NMR spectrum of compound 7

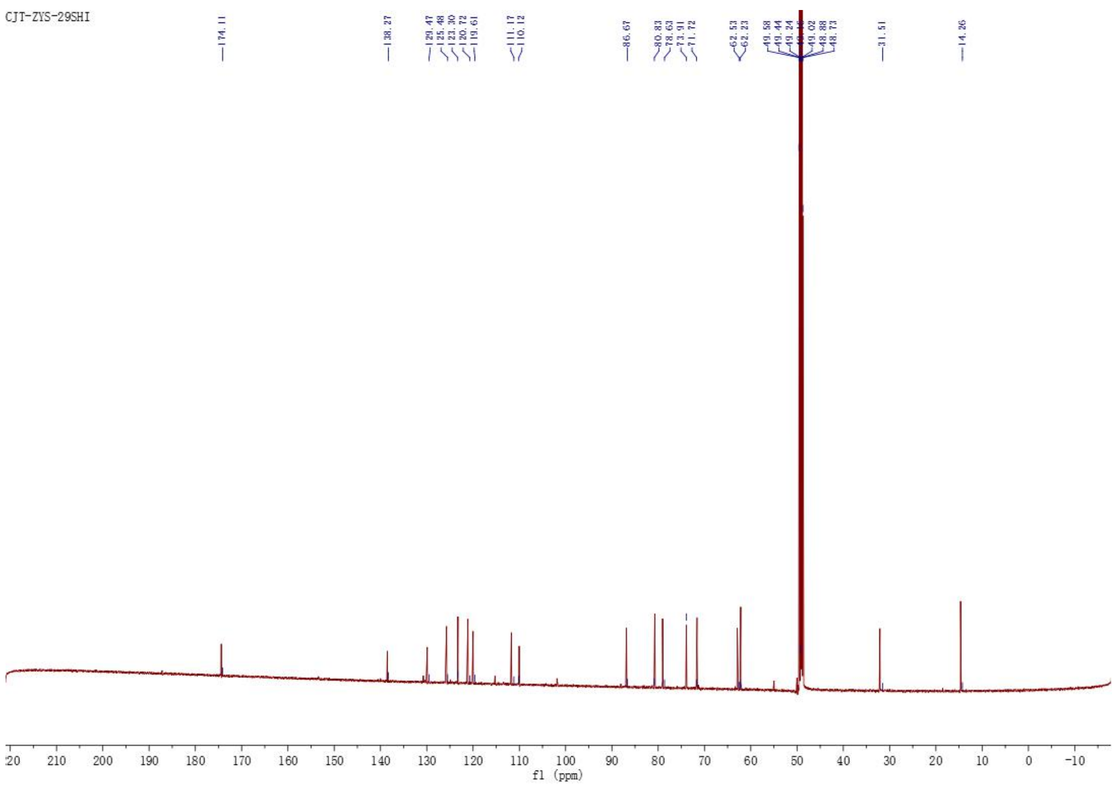

Figure S21. ^{13}**C NMR spectrum of compound 7**

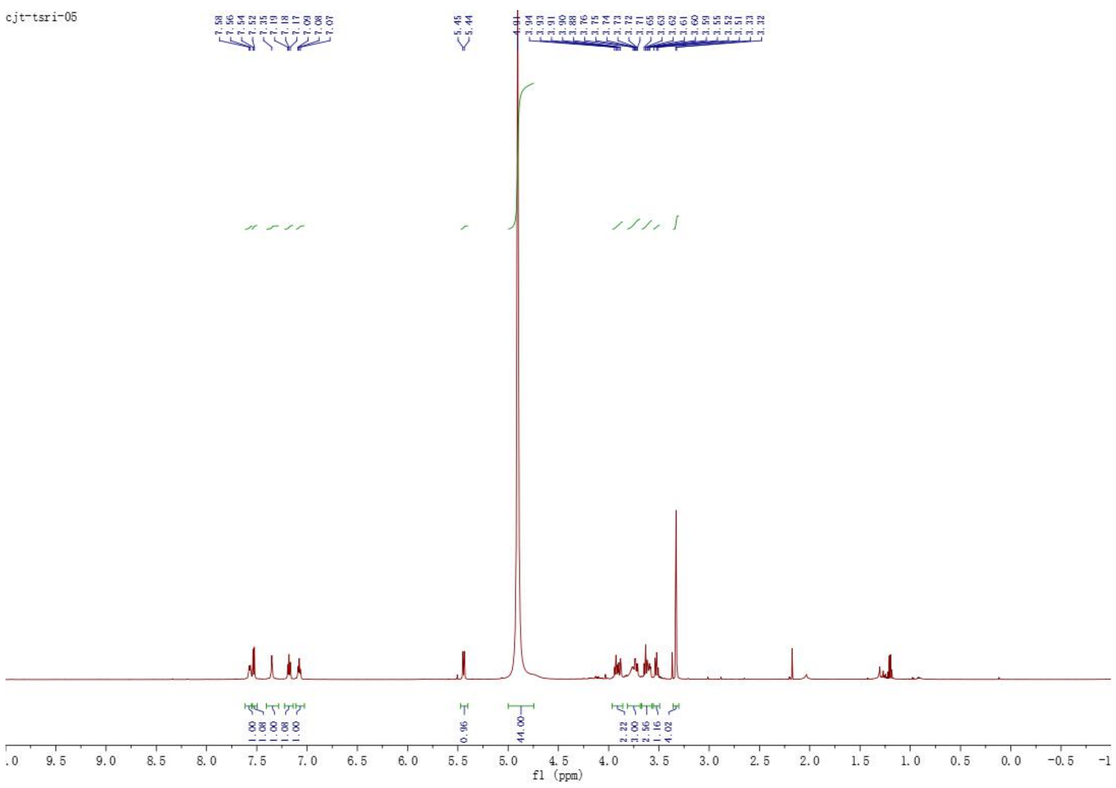

Figure S22. ^{1}H NMR spectrum of compound 8

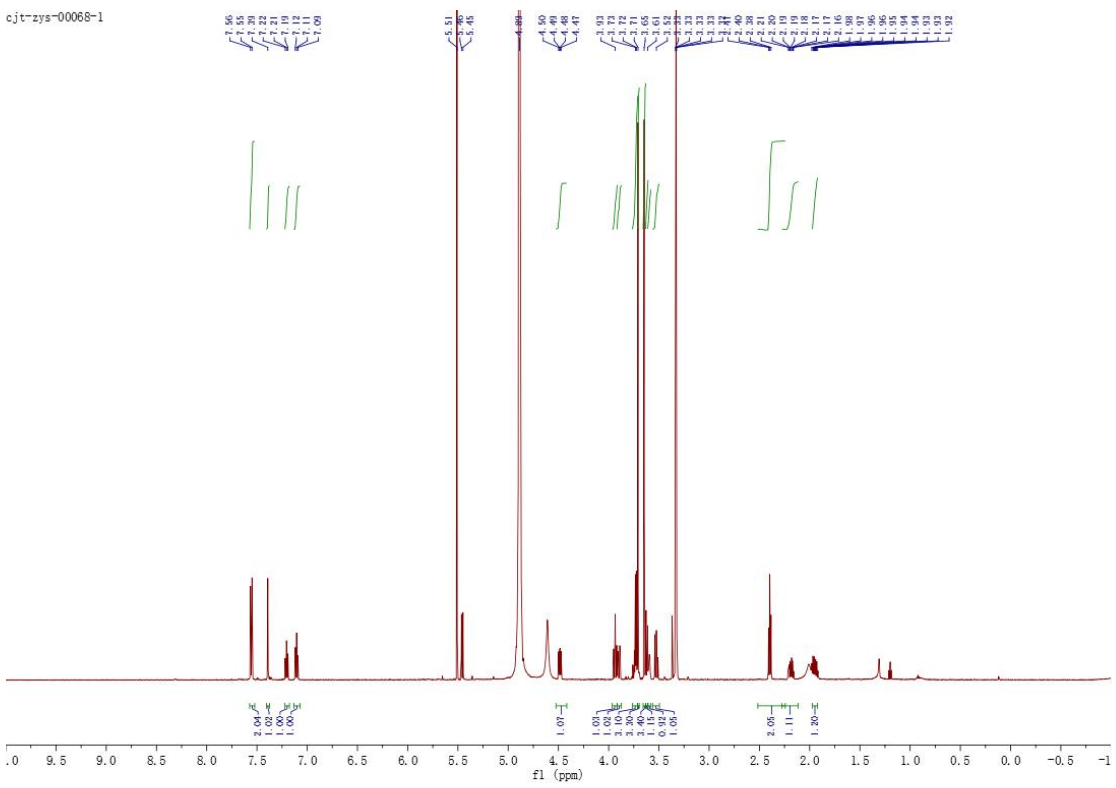

Figure S23. ^{1}H NMR spectrum of compound 9

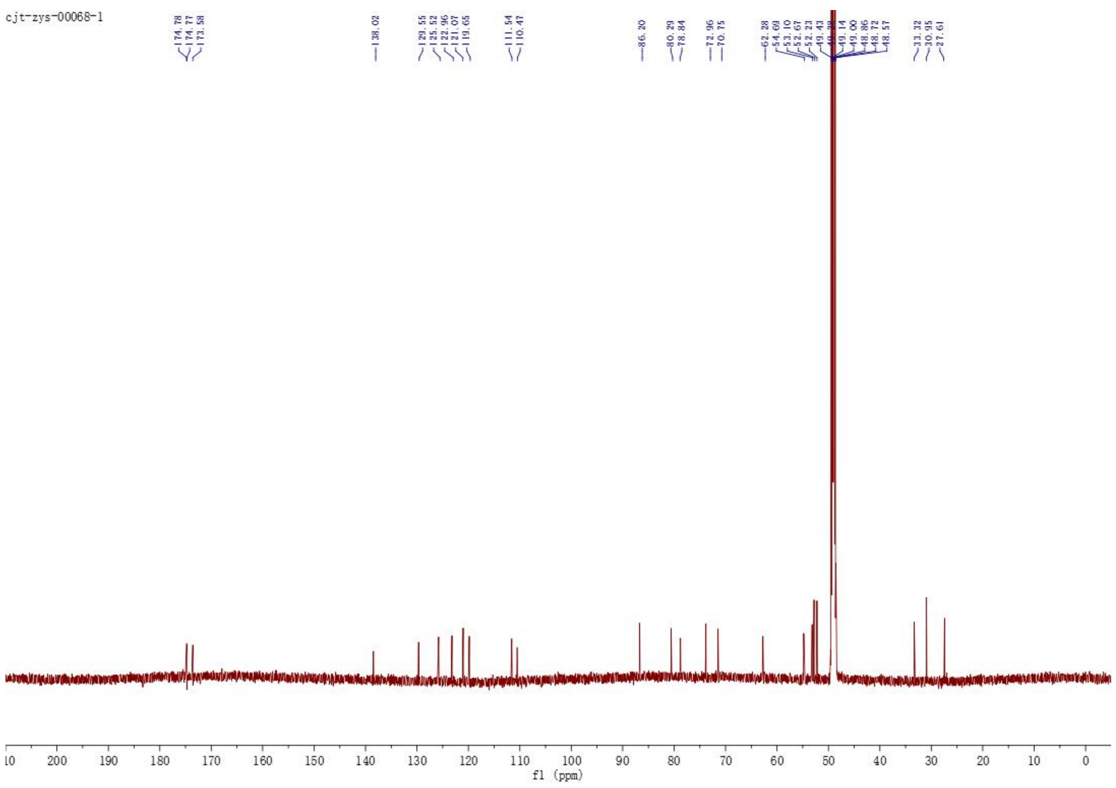

Figure S24. ^{13}C NMR spectrum of compound 9

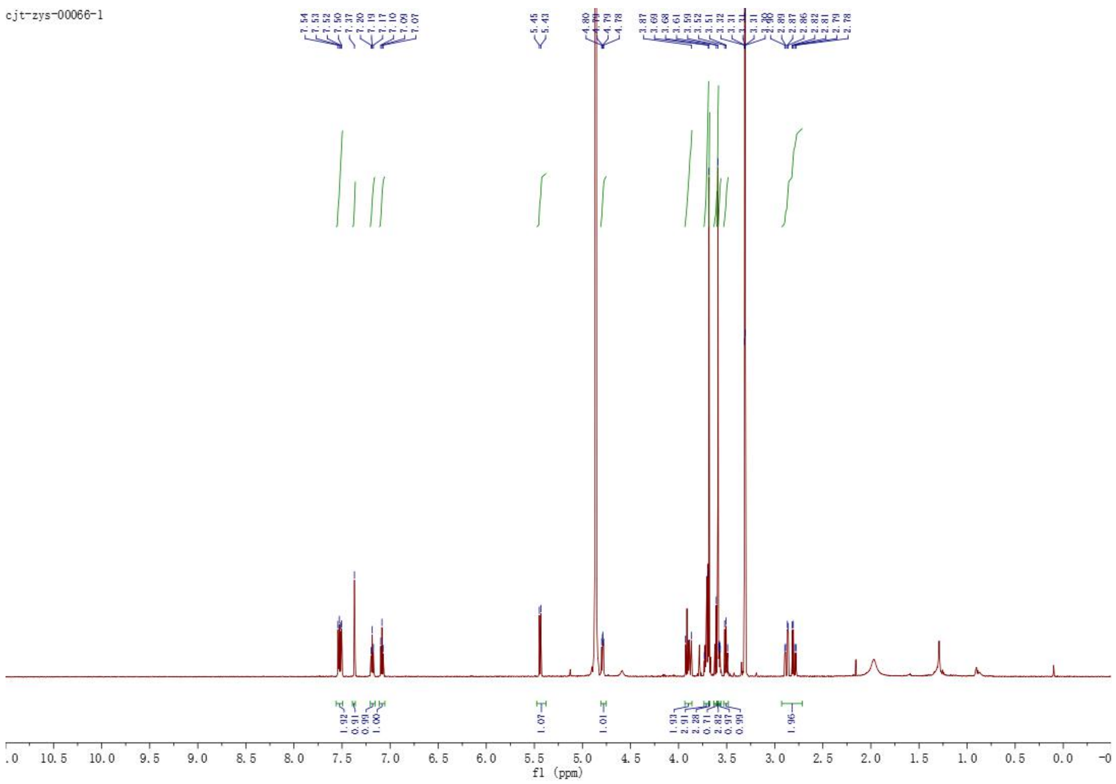

Figure S25. ¹H NMR spectrum of compound 10

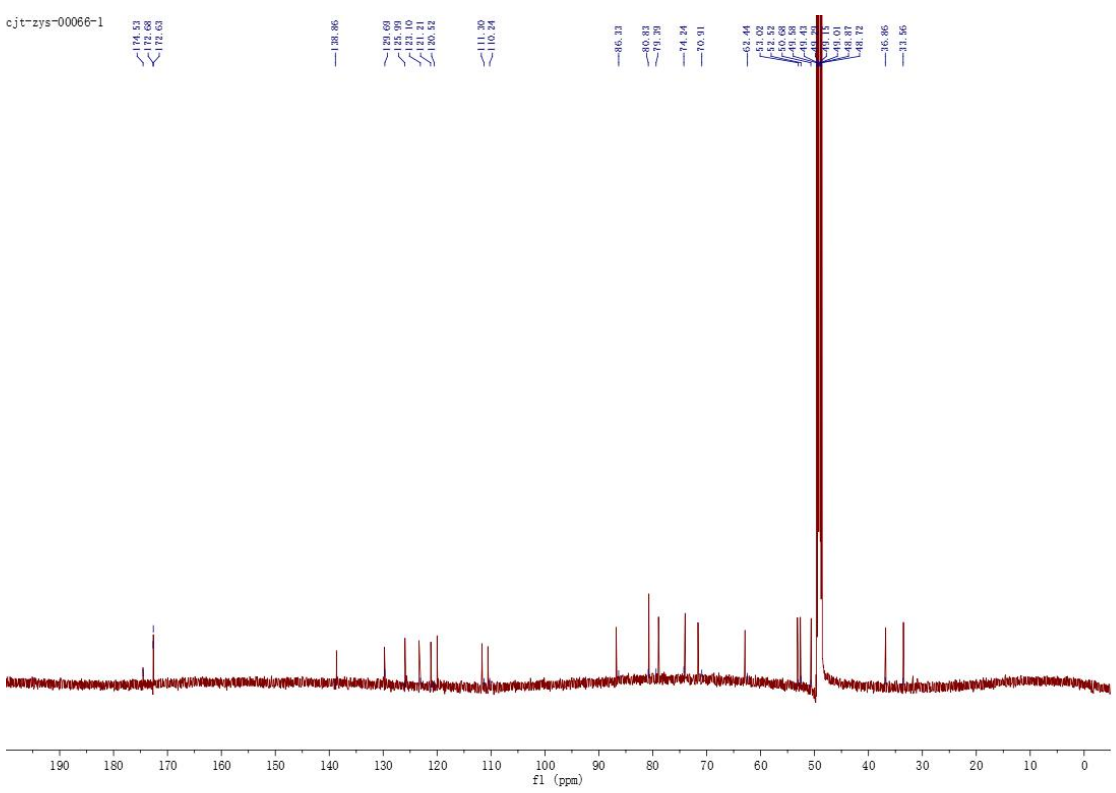

Figure S26. ^{13}C NMR spectrum of compound 10

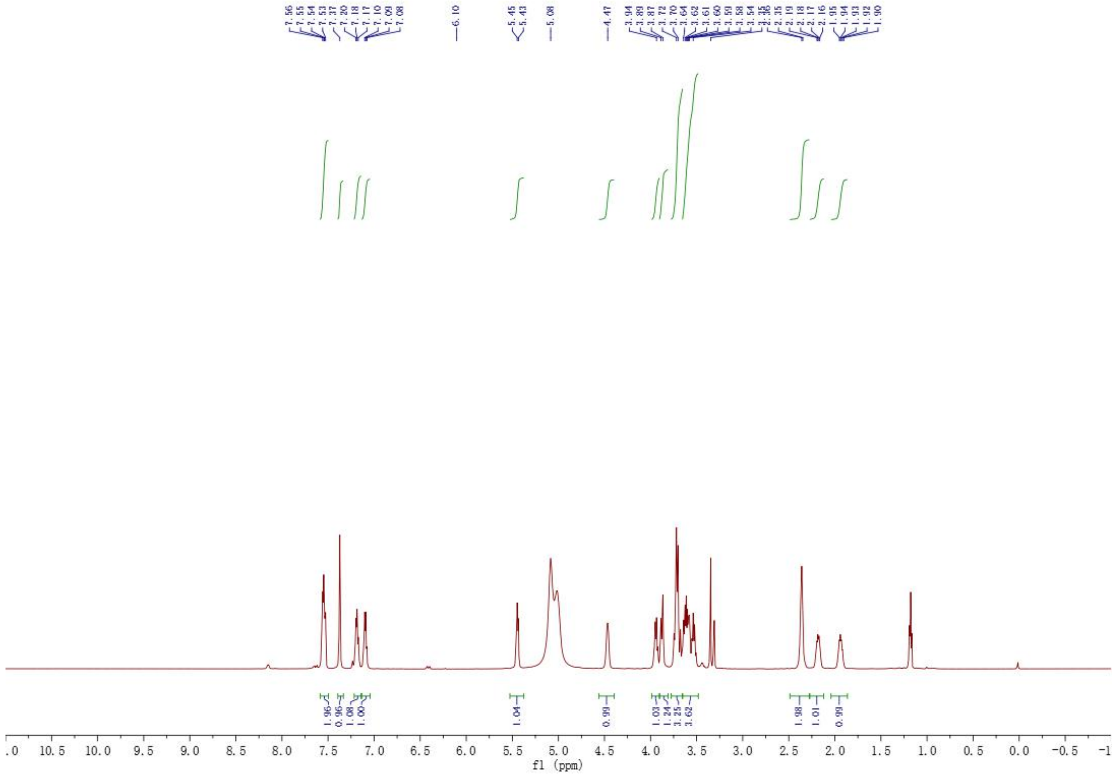

Figure S27. ¹H NMR spectrum of compound 11

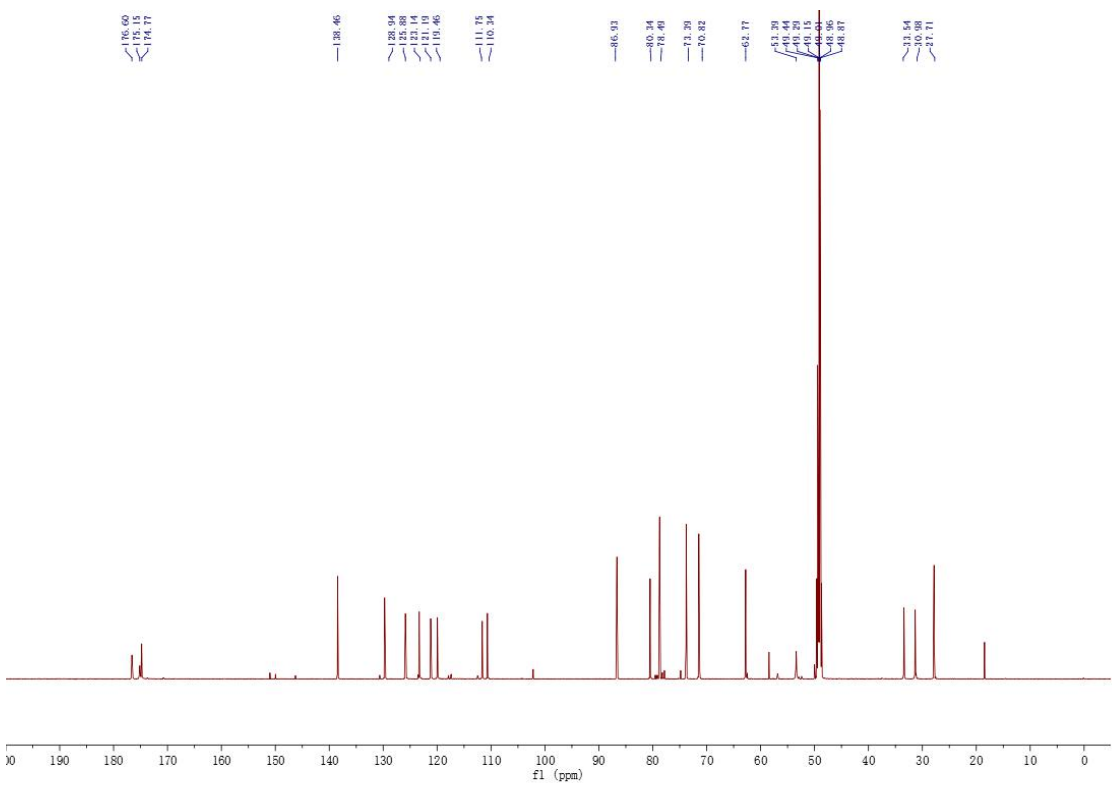

Figure S28. ^{13}C NMR spectrum of compound 11

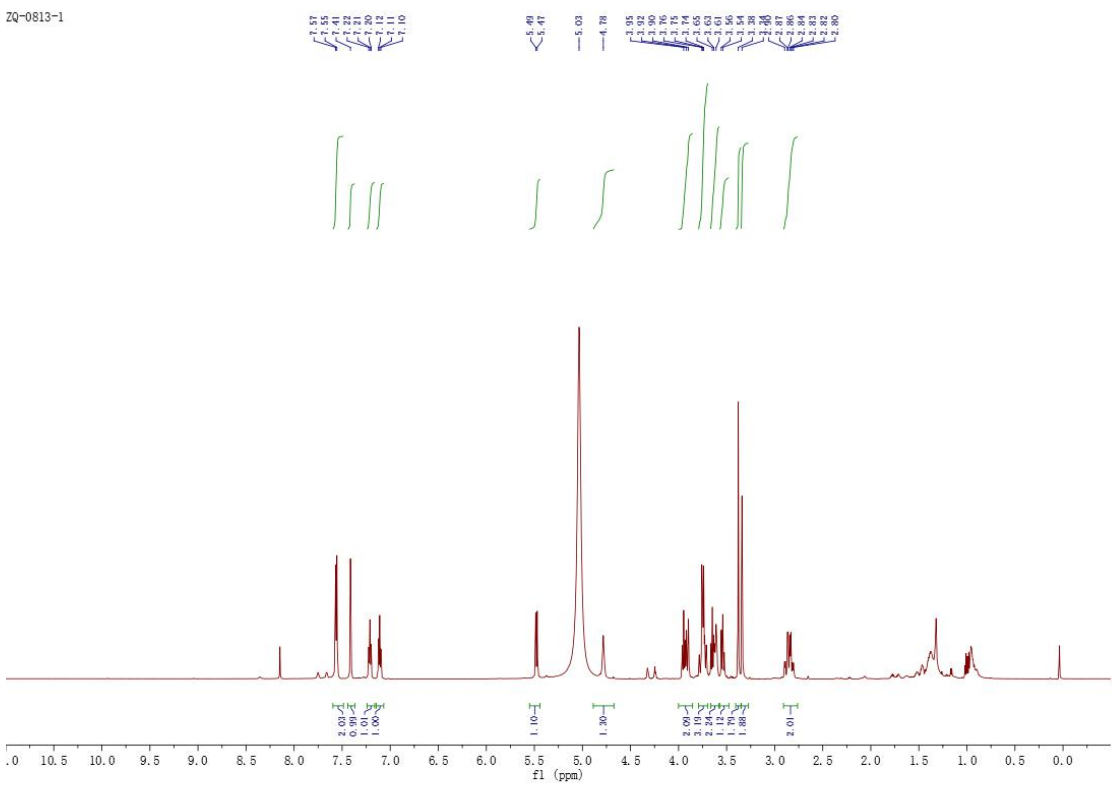

Figure S29. ^{1}H NMR spectrum of compound 12

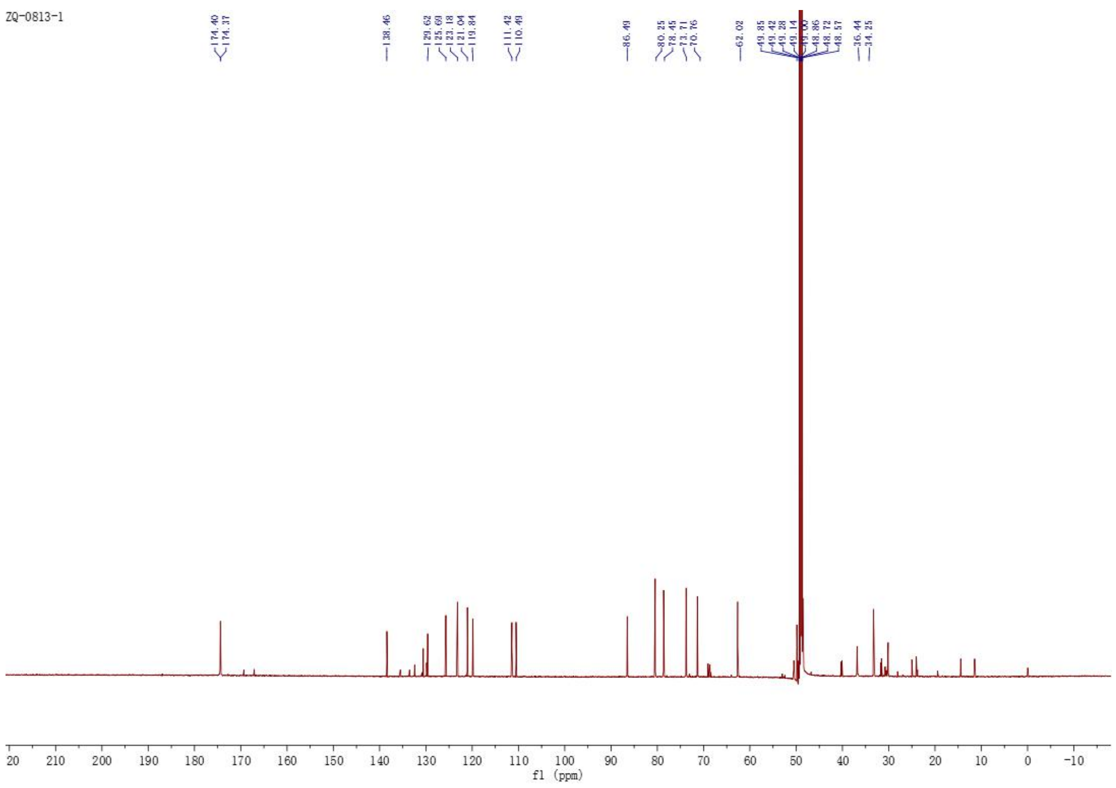

Figure S30. ^{13}C NMR spectrum of compound 12
